# Best Practices for Chiropractic Management of Patients with Chronic Musculoskeletal Pain: A Clinical Practice Guideline

**DOI:** 10.1089/acm.2020.0181

**Published:** 2020-10-08

**Authors:** Cheryl Hawk, Wayne Whalen, Ronald J. Farabaugh, Clinton J. Daniels, Amy L. Minkalis, David N. Taylor, Derek Anderson, Kristian Anderson, Louis S. Crivelli, Morgan Cark, Elizabeth Barlow, David Paris, Richard Sarnat, John Weeks

**Affiliations:** ^1^ Texas Chiropractic College, Pasadena, TX, USA.; ^2^ Private Practice, Santee, CA, USA.; ^3^ Advanced Medicine Integration Group, L.P., Columbus, OH, USA.; ^4^ VA Puget Sound Health Care System, Tacoma, WA, USA.; ^5^ Palmer Center for Chiropractic Research, Davenport, IA, USA.; ^6^ Texas Chiropractic College, Pasadena, TX, USA.; ^7^ Private Practice, Grand Forks, ND, USA.; ^8^ Private Practice, Greenbelt, MD, USA.; ^9^ Private Practice, Eureka, CA, USA.; ^10^ Texas Chiropractic College, Pasadena, TX, USA.; ^11^ VA Northern CA Health Care System, Redding, CA, USA.; ^12^ Seattle, WA, USA.

**Keywords:** chronic pain, chronic musculoskeletal pain, spinal manipulation, chiropractic, clinical practice guideline

## Abstract

***Objective:*** To develop an evidence-based clinical practice guideline (CPG) through a broad-based consensus process on best practices for chiropractic management of patients with chronic musculoskeletal (MSK) pain.

***Design:*** CPG based on evidence-based recommendations of a panel of experts in chronic MSK pain management.

***Methods:*** Using systematic reviews identified in an initial literature search, a steering committee of experts in research and management of patients with chronic MSK pain drafted a set of recommendations. Additional supportive literature was identified to supplement gaps in the evidence base. A multidisciplinary panel of experienced practitioners and educators rated the recommendations through a formal Delphi consensus process using the RAND Corporation/University of California, Los Angeles, methodology.

***Results:*** The Delphi process was conducted January–February 2020. The 62-member Delphi panel reached consensus on chiropractic management of five common chronic MSK pain conditions: low-back pain (LBP), neck pain, tension headache, osteoarthritis (knee and hip), and fibromyalgia. Recommendations were made for nonpharmacological treatments, including acupuncture, spinal manipulation/mobilization, and other manual therapy; modalities such as low-level laser and interferential current; exercise, including yoga; mind–body interventions, including mindfulness meditation and cognitive behavior therapy; and lifestyle modifications such as diet and tobacco cessation. Recommendations covered many aspects of the clinical encounter, from informed consent through diagnosis, assessment, treatment planning and implementation, and concurrent management and referral. Appropriate referral and comanagement were emphasized.

***Conclusions:*** These evidence-based recommendations for a variety of conservative treatment approaches to the management of common chronic MSK pain conditions may advance consistency of care, foster collaboration between provider groups, and thereby improve patient outcomes.

## Introduction

Pain prevalence has increased among United States adults by 25% from 1998 to 2014, according to a 2019 report, with 41% reporting pain in the period 2013–2014.^1^ At least 70 million U.S. adults have chronic pain.^1,2^ Opioid use has risen along with the increase in pain prevalence.^1^ Visits to health care providers decreased slightly within this same time period, perhaps suggesting that people tend to manage pain with medications rather than provider-based nonpharmacological approaches.^1^

Authoritative groups, including the Agency for Healthcare Research and Quality (AHRQ) and the American College of Physicians (ACP), have recommended that chronic back pain and other chronic musculoskeletal (MSK) pain be treated initially through nonpharmacological approaches.^3^

Some experts recommend viewing chronic pain as “a disease entity in itself, rather than merely a symptom of another condition.”^4^ The International Classification of Disease 11 (ICD-11) has created a new category of “chronic pain,” with the following disorders included: (1) chronic primary pain, which includes disorders such as fibromyalgia or back pain, which is not otherwise classified; (2) chronic cancer pain; (3) chronic post-traumatic and postsurgical pain; (4) chronic neuropathic pain; (5) chronic headache and orofacial pain, which includes temporomandibular joint pain; (6) chronic visceral pain; and (7) chronic MSK pain.^5,6^

The AHRQ, Institute of Medicine (IOM), and the National Pain Strategy Report^6–8^ recommend that chronic pain be addressed through the biopsychosocial model, rather than solely through the conventional biomedical model. This includes an emphasis on nonpharmacological and self-management approaches, with pharmacological approaches being secondary.^3,6–8^

The 2018 and 2020 AHRQ systematic reviews recommend noninvasive, nonpharmacological approaches to several of the most common chronic MSK pain conditions: chronic LBP (CLBP), chronic neck pain, osteoarthritis (OA), fibromyalgia, and chronic tension headache.^6,9^ A 2018 review in the *Journal of Family Practice* organized its evidence-based recommendations for common chronic pain conditions by the treatment approach: (1) exercise-based therapies such as yoga and *t'ai chi*; (2) mind–body therapies such as Cognitive Behavioral Therapy (CBT) and mindfulness-based meditation; and (3) complementary modalities such as acupuncture and spinal manipulation.^10^

The purpose of this project was to develop a clinical practice guideline (CPG) for chiropractic management of chronic MSK pain. The chiropractic profession's primary approach to patient care has traditionally been spinal manipulation, but its scope of practice includes many other nonpharmacological approaches.^11^ Like medical physicians, chiropractors may not be familiar with many of these approaches other than spinal manipulation, or may not directly employ them with patients. It is important that all health care providers become familiar with evidence-based approaches, within a biopsychosocial model, to help patients manage chronic pain. This is important whether the provider directly employs such approaches, refers the patient to other providers who do, or advises the patient on self-care activities.

In response to the opioid epidemic, nonpharmacological approaches to chronic pain management are expected to become increasingly legitimized.^12^ Because the public expects Doctors of Chiropractics (DCs) to use such therapies more than medical physicians do, they may be more likely to seek out chiropractic practitioners for these therapies.^13^ Thus it is important that DCs become familiar with these approaches within the context of the biopsychosocial model. Currently, although there are CPGs addressing a chiropractic approach to LBP,^14,15^ neck pain,^16,17^ and headaches^18^ separately, there is not a single CPG addressing nonpharmacological approaches to more than one type of MSK pain as a primary complaint. The purpose of this project was therefore to develop such a guideline.

## Methods

The purpose of the project was to develop an evidence-based CPG through a broad-based consensus process on best practices for chiropractic management of patients with chronic MSK pain.

The development of recommendations followed steps developed and tested in previous projects^15,17,19^:

- Establish a Steering Committee (SC) to perform the core project functions of examining the evidence, developing recommendations based on the best available evidence, and integrating the Delphi panelists' ratings and contributions into the recommendations until a consensus is reached.
- Examine the most current CPGs and/or systematic reviews related to each aspect of management.
- Identify gaps in the CPG(s) and/or systematic reviews that may form barriers to best practices.
- Perform targeted literature searches for the highest available evidence on the gap topics.
- Make recommendations on chiropractic management, based on the best available evidence.
- Conduct a Delphi consensus process with a panel of practitioners, faculty, and researchers experienced in chronic MSK pain management.
- Gather additional feedback from a public posting of the consensus statements.^15^

### Human subject considerations

The lead institution's Institutional Review Board approved the project before it started. All Delphi panelists participated voluntarily and without compensation; they signed an informed consent and agreed to be acknowledged by name in any publication only if they signed a consent to be acknowledged.

### Project SC

Of the 11-member SC, 8 were DCs. All of these have extensive experience in chiropractic management of chronic MSK pain and/or knowledge of the evidence base on clinical care of MSK pain. All have held or currently hold leadership positions in chiropractic professional organizations, education and/or research. Three of the DCs are members of the Scientific Council of the Clinical Compass (Council on Chiropractic Guidelines and Practice Parameters. Three of the DCs work full time at the Veterans Health Administration (VA); two are full-time faculty at chiropractic institutions; and one DC is cross-trained as a registered nurse (RN). The project director is a DC with a PhD in Preventive Medicine and is also a Certified Health Education Specialist. One SC member is a medical physician (MD) with many years of experience with chronic pain management; one is a psychologist (PhD) who works with chronic pain patients in the VA; and one is a representative for laypeople and also a journal editor with extensive experience with complementary health care. The SC was responsible for identifying, reviewing, and evaluating the evidence underlying the development of the initial seed statements, modifying these statements based on the Delphi panelists' comments, and writing the final article.

### Literature search

The literature search focused on the evidence base for nonpharmacological, nonsurgical interventions for chronic MSK pain. A health sciences librarian, working with the SC, conducted the literature search in two stages. The databases we searched were Cochrane Database of Systematic Reviews and PubMed/Medline, because it is unlikely that higher levels of evidence would be found in other databases, but not in these. The search strategy may be accessed in Supplementary Data S1. In addition, we used reference tracking and consulted topic experts on the SC to ensure that relevant articles were not missed.

#### First stage search

To identify a “seed” document or documents on which to base development of the initial set of recommendations, we conducted two searches: (1) identify the most recent systematic reviews for nonpharmacological treatment of chronic MSK pain and (2) identify CPGs specific to manipulation and manual therapy. We restricted the searches to recent literature rather than doing a comprehensive search, since CPGs should be based on the most current literature, and current systematic reviews were expected to cover earlier studies.^20^

*Search 1 inclusion criteria*:

1. Published January 1, 2017, to August 15, 2019.
2. English language.
3. Addressed nondrug, nonsurgical treatment of chronic MSK pain in adults.
4. Systematic reviews/meta-analyses.

Exclusion criteria:

1. Nonrelevant (e.g., addressed interventions outside the scope of U.S. chiropractors or addressed risk factors, but not interventions; did not address chronic MSK pain).
2. Addressed only one type of MSK pain as a primary complaint (e.g., only back pain) and/or one type of intervention (e.g., only CBT), to have a comprehensive seed document to base our recommendations.
3. Included in another systematic review.

Search 2 inclusion criteria

1. Guidelines related to spinal manipulation and/or manual therapy.
2. Published 2016–2019.
3. English language.

Exclusion criteria:

1. Nonrelevant (not CPGs; outside chiropractic scope of practice or not related to chronic MSK pain).

#### Second stage search

First, we drafted preliminary evidence-based recommendations based on the results of the initial search. In cases where recommendations for specific modalities or procedures were absent due to sparse evidence for procedures commonly used in chiropractic practice (as identified by the current *Practice Analysis of Chiropractic*^11^), we did a targeted search of the published literature from the end date of the source systematic review or guideline through 2019. We included guidelines, systematic reviews, randomized controlled trials, or outcome cohort studies.

### Evaluation of the quality of the evidence

We then evaluated the quality of the articles identified in our searches. We evaluated CPGs using the Appraisal of Guidelines for Research & Evaluation instrument (AGREE) Global Rating Scale (Table 1).^21^ We evaluated systematic reviews, RCTs, and cohort studies investigating treatments using modified SIGN (Scottish Intercollegiate Guideline Network) checklists, which have been used in other studies by our team.^22–24^ The SIGN checklist rates the studies as “high quality, low risk of bias,” “acceptable quality, moderate risk of bias,” “low quality, high risk of bias,” or “unacceptable” quality. See Tables 2–4 for details of scoring. We did not assess the quality of other types of studies, simply identifying their design and categorizing them as “lower level.” At least two investigators rated each study and discussed differences in ratings until they reached agreement.

**Table 1.** AGREE Global Rating Scale

Each item is rated on a 1–7 scale from lowest (1) to highest (7) quality; maximum score = 49. Quality assessed as follows:
• Divide total score by 7 for average score.
• High quality: average 6–7; acceptable quality: average 4–5; unacceptable quality: <4
Process of development
1. Rate the overall quality of the guideline development methods.
• Were the appropriate stakeholders involved in the development of the guideline?
• Was the evidentiary base developed systematically?
• Were recommendations consistent with the literature
Presentation style
2. Rate the overall quality of the guideline presentation.
• Was the guideline well organized?
• Were the recommendations easy to find?
Completeness of reporting
3. Rate the completeness of reporting.
• Was the guideline development process transparent and reproducible?
• How complete was the information to inform decision-making?
Clinical validity
4. Rate the overall quality of the guideline recommendations.
• Are the recommendations clinically sound?
• Are the recommendations appropriate for the intended patients?
Overall assessment
5. Rate the overall quality of this guideline.
6. I would recommend this guideline for use in practice.
7. I would make use of a guideline of this quality in my professional decisions.

**Table 2.** Randomized Controlled Trial Modified SIGN Checklist

	Item	Yes/no^a^
1	The study addressed an appropriate and clearly focused question.	
2	Group assignment was randomized.	
3	The sample size was justified by a power calculation.	
4	Investigators were blinded to patients' group assignment.	
5	Patients were blinded to group assignment.	
6	Groups were similar at the start of the trial.	
7	The only difference between groups was the treatment of interest.	
8	Outcomes were measured in a standard, valid, and reliable way.	
9	A power calculation was used and required sample size attained.	
10	An intention to treat analysis was performed.	
	Total score^b^	

^a^ Rating: “Yes” = 1; “No” or unable to tell from the article = 0.

^b^ Scoring—sum of items as follows: 9–10 = high quality, low risk of bias; 6–8 = acceptable quality, moderate risk of bias; <6 = low quality, high risk of bias.

**Table 3.** Cohort Study Modified SIGN Checklist

	Item	Yes/no^a^
1	Addresses an appropriate and clearly focused question.	
2	Groups are similar, except for factor of interest.	
3	Number of people who declined enrollment is stated.	
4	Likelihood that some patients might have the outcome when enrolled are taken into account in the analysis.	
5	Attrition in each group stated.	
6	Dropouts and compliant participants compared by exposure.	
7	The outcomes are clearly defined.	
8	Assessment of outcome is made blind to exposure status.	
9	The method of assessment of exposure is reliable.	
10	Evidence from other sources is used to demonstrate that the method of outcome assessment is valid and reliable.	
11	Main potential confounders identified and accounted for in design and analysis.	
12	Confidence intervals are reported.	
	Total score^b^	

^a^ Rating: “Yes” = 1; “No” or unable to tell from the article = 0.

^b^ Scoring—sum of items as follows: 10–12 = high quality, low risk of bias; 6–9 = acceptable quality, moderate risk of bias; <6 = low quality, high risk of bias.

**Table 4.** Systematic Review/Meta-Analysis Modified SIGN Checklist

	Item	Yes/no^a^
1	Research question was clearly defined and eligibility criteria listed.	
2	A comprehensive literature search was conducted.	
3	At least two people selected studies.	
4	At least two people extracted data.	
5	The status of publication was not used as an inclusion criterion.	
6	The excluded studies were listed.	
7	The relevant characteristics of included studies were provided.	
8	The quality of included studies was assessed and reported.	
9	At least two people assessed quality of the included studies.	
10	Appropriate methods were used to combine individual study results.	
11	Likelihood of publication bias was assessed appropriately.	
12	Conflicts of interest were declared.	
	Total score^b^	

^a^ Rating: “Yes” = 1; “No” or unable to tell from the article = 0.

^b^ Scoring—sum of items as follows: 10–12 = high quality, low risk of bias; 6–9 = acceptable quality, moderate risk of bias; <6 = low quality, high risk of bias.

We used the GRADE (Grading of Recommendations Assessment, Development, and Evaluation) system to assess the overall quality of the evidence.^25,^^*^ Table 5 summarizes GRADE.^25^ At least two investigators performed the GRADE assessment independently. If they disagreed, they discussed the assessment and used the majority opinion.

**Table 5.** Rating the Quality of Evidence Using the Grading of Recommendations Assessment, Development and Evaluation System

Level of evidence	Quality rating	Explanation of quality rating
A	High	High level of confidence in the effects of the intervention.
		• Several high-quality studies with consistent outcomes
B	Moderate	Confidence in the effects of the intervention may change with future research findings
		• Only one high-quality study *or*
		• Several lower quality studies
C	Low	Confidence in the effects of the intervention is very likely to change with future research findings
		• All studies have severe limitations
D	Very Low	Uncertainty about the effects of the intervention
		• Only expert opinion *and/or*
		• No research evidence *or*
		• Very low-quality evidence

Source: GRADE.^103^

### Development of seed statements

The SC drafted a set of seed statements/concepts encompassing key aspects of the clinical encounter, including informed consent, diagnosis, treatment, concurrent care and co-management, and/or referral. Based on the literature, in addition to statements regarding chronic MSK pain in general, we addressed five of the most common chronic MSK pain conditions: LBP, neck pain, knee and hip OA pain, and fibromyalgia.^6^ We cited evidence supporting all statements in the text and provided live links to the full text or abstracts in the attached reference list, so that during the consensus process, panelists could conveniently access them to make an evidence-informed rating.

### Delphi consensus panel

We sought to recruit a broad-based panel of DCs and other health professionals who had experience with managing patients with chronic MSK pain, valued scientific evidence, and were geographically dispersed throughout the United States. We focused on the United States because practice parameters and reimbursement issues vary among countries. We also made it clear to participants that they must be able to respond in a timely manner to the process, which was conducted by e-mail.

We recruited Delphi panelists by (1) inviting experts who had participated in our previous consensus projects and (2) circulating an invitation through the Clinical Compass board, which includes representatives of the Congress of Chiropractic State Associations, the American Chiropractic Association, the International Chiropractors Association, and the Association of Chiropractic Colleges. The SC reviewed the resulting volunteers, who submitted both a form with their practice characteristics and their CV.

### Methodology of the Delphi process

The process was conducted electronically, through e-mail. Throughout the process, panelists remained anonymous, having been assigned an identification number at the beginning. This was done to avoid possible bias, since all raters' comments were shared among the SC and the Delphi panelists. As in all of our previous consensus processes, we used the RAND-UCLA methodology.^26^ This method employs an ordinal Likert “appropriateness” rating scale in which “appropriate” indicates that the expected patient health benefits exceed expected negative effects by a large enough margin that the recommended action is worthwhile, without considering costs.^26^ This 1–9 scale is anchored by 1 = “highly inappropriate and 9 = “highly appropriate, with “uncertain” placed over the middle of the scale. Panelists had unlimited space for comments immediately following each statement. They were also instructed to provide citations to support their comments, if possible.

#### Data management and analysis

The project coordinator entered the ratings data into an SPSS (v. 25) database, and she and the project director computed medians and percentages of agreement. In keeping with the rigorous RAND-UCLA methodology, we set the threshold for consensus at 80% agreement with a median rating of at least seven. This was calculated by categorizing ratings of 1–3 as “inappropriate” (i.e., disagreement with the statement); 4–6 as “uncertain”; and 7–9 as “appropriate” (i.e., agreement). The project coordinator organized the panelists' comments by panelist ID, statement number, and rating to facilitate review. The SC then reviewed the ratings and their accompanying deidentified comments. Taking the comments and supporting evidence into account, the SC then revised the statements that did not reach consensus. The project coordinator provided these revised statements and the deidentified comments to the Delphi panel for another round of rating.

### External review: Public comments

Influential organizations such as the AGREE Enterprise recommend incorporating various means for ensuring stakeholder involvement into a guideline development process. We already involved stakeholders in the SC and the Delphi panel. For additional input, we invited public comments on the draft CPG after completing the Delphi process. We used several routes to disseminate this invitation:

- Clinical Compass e-mailing list through a MailChimp e-mail blast; this includes the Clinical Compass Board (comprised United States state chiropractic organizations and a number of national chiropractic and academic organizations (about 900 individuals total). It also includes vendors, whose contacts included interested laypersons.
- Invitations were sent through the chiropractic organization ChiroCongress to its member associations, representing over 35,000 chiropractors.
- Facebook and LinkedIn through the Clinical Compass page, which is open to both health professionals and interested laypersons
- Chiropractic Summit e-mail list; this is a national organization of chiropractic groups and individuals.

These routes had some overlap, which served to reinforce the message. In addition, a reminder was sent out 2 weeks after the first invitation. We allowed 30 days for the comment period.

We posted the draft CPG on the Clinical Compass website as a PDF, along with a summary of the background and methodology of the project, as well as the references for all statements. We provided a user-friendly comment form to facilitate response. The project coordinator collected responses. The project director and the SC reviewed and decided how to respond to each comment. If the comments resulted in substantive change, the revised statements were to be recirculated to the Delphi panel to reach consensus.

## Results

### Literature search and evaluation

#### First stage search 1: Systematic reviews

**FIG. 1.**
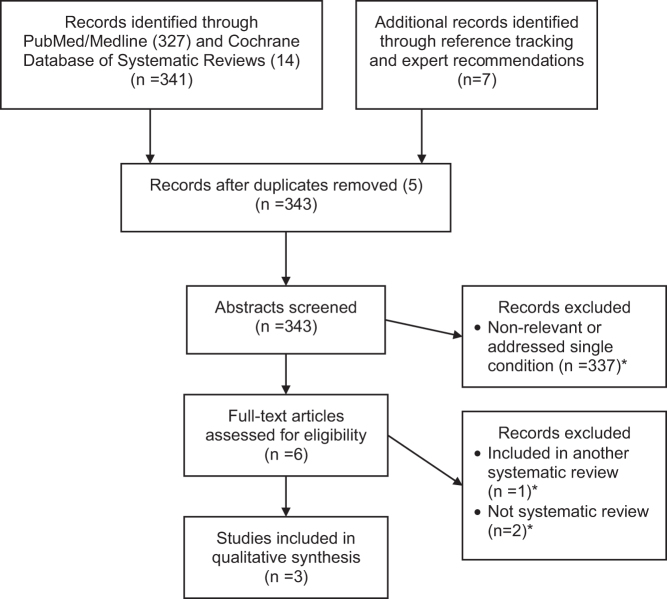
PRISMA flow diagram for first-stage literature search. Excluded studies listed in Supplementary Data.

We identified 343 articles (guidelines and systematic reviews/meta-analyses) through PubMed, Cochrane Database of Systematic Reviews, reference tracking, and consultation. Figure 1 is the Preferred Reporting Items for Systematic Reviews and Meta-Analyses (PRISMA) flow chart for the literature search. After applying eligibility criteria, three systematic reviews remained.^6,10,27^ (Excluded articles are available in Supplementary Data S2.)

##### Evaluation

We evaluated two of the articles as high quality^6,27^ and one as unacceptable quality^10^; we did not use the unacceptable (low) quality study to support recommendations. We selected one of the two remaining articles, the extensive and high-quality systematic review by the AHRQ on noninvasive nonpharmacological treatment for chronic pain,^6^ as an appropriate document to serve as the initial framework for our recommendations. We accepted AHRQ's overall rating of the quality of evidence for noninvasive, nonpharmacological interventions as low to moderate and that “there was no evidence suggesting increased risk for serious treatment-related harms for any of the interventions, although data on harms were limited.”^6,p.ii^ We included in our CPG, the five conditions covered in the AHRQ review, which are among the most common causes of chronic MSK pain: LBP, neck pain, chronic tension headache, OA (knee and hip), and fibromyalgia.^6^

#### First stage search 2: Clinical practice guidelines

From an initial pool of 147 articles, 23 remained after title screening and 10 remained after abstract/full-text screening. Table 6 lists these CPGs; all were considered high quality, either by our rating with AGREE or a published systematic review of the quality of CPGs on MSK pain using AGREE.^28^ All the guidelines were single-condition focused: 5 on neck pain,^16–18,29,30^ 4 on LBP,^3,14,15,31^ and 1 on headaches associated with neck pain.^32^ There were none on other types of chronic MSK pain.

**Table 6.** Clinical Practice Guidelines That Include Manipulation and Manual Therapies, 2016–2019

Topic	First author	Year	Quality^a^
Chronic headache associated with neck pain	Cote.^32^	2019	H
Acute and CLBP	Globe^15^	2016	H^b^
Acute and CLBP	Bussieres^14^	2018	H
Acute and CLBP and sciatica	National Guideline Center^31^	2016	H
Acute and CLBP	Qaseem^3^	2017	H
Acute and chronic neck pain	Whalen^17^	2019	H
Acute and chronic neck pain	Cote^18^	2016	H^b^
Acute and chronic neck pain	Blanpied^30^	2017	H
Acute and chronic neck pain	Bussieres^16^	2016	H
Acute and chronic neck pain	Bier^29^	2018	H

^a^ Quality was assessed using the AGREE Global Rating Scale (Table 4).

^b^ Rating from a published review of CPGs related to musculoskeletal pain.^28^

CLBP, chronic low-back pain.

#### Second stage search

We did a targeted search of the published literature from the end date of the AHRQ review (November 1, 2017) for topics that showed gaps in the evidence for therapies used commonly in chiropractic practice. The interventions we performed searches for were spinal manipulation/manual therapy, transcutaneous electrical nerve stimulation (TENS) and interferential current, low-level laser (LLL) therapy, and acupuncture. Table 7 summarizes the articles identified after searching for each specific modality from a pool of 348 articles. There were a total of 21 articles: 5 CPGs,^14,17,29,32,33^ 4 RCTS,^34–37^ and 12 SRs,^38–49^ as shown by condition and therapy in Table 7. Nine were acceptable quality and 11 were high quality, using the modified SIGN rating checklists shown in Tables 1–3 or, for CPGs, the AGREE scale shown in Table 4).

**Table 7.** Evidence from Targeted Search for Interventions 2018–2019, by Condition

Condition	Design	First author	Quality	Primary intervention
LBP	CPG	Bussieres^14^	H	SM/MT
	SR	Wu^47^	H	TENS
	SR	Almeida^38^	A	TENS/IFC
	RCT	Barone-Gibbs^35^	A	MB/L
	RCT	Eklund^36^	H	SM
Neck pain	CPG	Whalen^17^	H	SM/MT
	CPG	Bier^29^	H	SM/MT
	SR	Almeida^38^	A	TENS/IFC
	RCT	Albornoz-Cabello^34^	H	IFC
	RCT	Yesil^37^	A	TENS/IFC
Headache	CPG	Cote et al.^32^	H	SM/MT
	CPG	Steiner^33^	H	Multiple
	SR	Gu^42^	H	MB/L
Knee OA	SR	Gong^41^	A	ACU
	SR	Sun^46^	A	ACU
	SR	Stausholm^45^	H	LLL
	SR	Wysynska^48^	A	LLL
	SR	Anwer^39^	H	MT
Hip OA	SR	Ceballos-Laita^40^	H	MT
Fibromyalgia	SR	Kim^44^	H	ACU
	SR	Yeh^49^	A	LLL
	SR	Honda^43^	A	LLL/TENS

ACU, acupuncture; LLL, low-level laser therapy; MB/L, mind–body, psychological therapies or lifestyle counseling; MT, manual therapy; OA, osteoarthritis; SM, spinal manipulation; TENS/IFC, transcutaneous nerve stimulation/interferential current; Multiple = various nonpharmacological therapies, including those already listed and others.

Table 8 summarizes the quality of the evidence from both the AHRQ review and our targeted search (2018–2019). Overall, the evidence was favorable, moderate to low.

**Table 8.** Quality of Evidence for Targeted (Procedure/Topic Specific) Searches

	CLBP	CNP	CTTH	KOA	HOA	FM
SM/MT	***C***	B	***C***	B	***C****;* B	***C (MT)***
						C (SM)
TENS/IFC	B	C		B		C
LLL	***C***	***B***		B		B
ACU	***C***	***C***	***C***	***B-C;*** B		***C***
MB/L	***B-C***		B			***C***

Strength of evidence rated by the AHRQ systematic review is in ***bold italics***. Strength of evidence is otherwise based on rating of literature published 2018–2019, including clinical practice guidelines, systematic review/meta-analyses, and randomized controlled trials. GRADE classifications: (see Table 5 for details): A = high; B = moderate; C = low; D = very low.

ACU, acupuncture; CLBP, chronic low-back pain; CNP, chronic neck pain; CTTH, chronic tension-type headache; FM, fibromyalgia; HOA, hip osteoarthritis; KOA, knee osteoarthritis; LLL, low-level laser therapy; MB/L, mind–body, psychological therapies and lifestyle counseling; MT, manual therapy; SM/MT, spinal manipulation/manual therapy; SM, spinal manipulation; TENS/IFC, transcutaneous nerve stimulation/interferential current.

### Delphi process

There were 62 panelists (of 70 invited); 58 were DCs. Ten DCs were cross-trained: five in acupuncture, three in physical therapy (Doctor of Physical Therapy [DPT]), two in medicine (MD), two in nursing (RN), and one in mental health counseling (MA). Eighteen of the DCs had academic master's degrees. One panelist was an MD and three were DPTs. Almost all (57) were practitioners with an average time in practice of 24 years (range 1–48). Sixteen of the panelists worked in the Veterans Administration (VA) and one had a referral arrangement with a local VA. Seven panelists were faculty at chiropractic institutions and seven were faculty at nonchiropractic institutions. Practitioners saw an average of 82 patient visits per week (range: 12–250) and the average estimated proportion of patients with a chief complaint of chronic (>3 months' duration) MSK pain was 61% (range: 15–100). Panelists' locations (58 of 62 responded) represented 31 states plus 1 from Australia and 1 from Canada as follows: five from CA; four each from IA and NY; three each from AZ, KS, MI, OH, and TX; two each from MD, MN, MO, NY, OR, SD, an WA; and one each from CO, HI, IL IN, MA, MS, MT, NC, ND, PA, RI, SC, and TN.

On the first Delphi round, a high level of consensus (from 87% to 100% agreement) was reached on all statements. The panelists had extensive comments, but most were based on clarifying rather than substantively changing the statements. The SC made revisions for the purposes of clarification.

### Public comments

We disseminated an invitation for comment very widely through the Clinical Compass board, chiropractic state and national organizations, thus reaching the majority of chiropractors in the United States as well as interested laypeople. Postings on the organization's Facebook page and website were accessed by 209 different people. We received three public comments. All were from DC faculty at U.S. chiropractic colleges; their suggestions were detailed and specific, primarily recommending clarifications in the wording of statements. The SC reviewed their comments and made a number of nonsubstantive changes for clarity in the seed statements; additional Delphi rounds were therefore not required. The final statements are found below.

### Chronic pain terminology and definitions

Based on the literature, we prefaced the Delphi consensus process with definitions of key terminology so that panelists would be “on the same page” as they rated the statements.

#### Chronic pain terminology

- **Chronic pain:** persistent or recurrent pain lasting longer than 3 months (ICD-11 definition)^5^ or pain present on at least half the days during the past 6 months (National Pain Strategy definition).^8^
- **Chronic primary pain***:* chronic pain in one or more anatomic locations accompanied by significant emotional distress or functional disability and that cannot be better explained by another chronic pain condition.”^5^
- **High impact chronic pain:** chronic pain that causes enduring restrictions on activities of daily living, work, social, and/or recreational activities.^8^
- **Neuropathic pain** is identified using the following criteria^50,51^:

1. Confirmed pain distribution and sensory dysfunction that are neuroanatomically congruent.
2. Confirmed history or presence of a relevant disease or lesion affecting the peripheral or central nervous system.
3. A description of burning, shooting, or pricking pain.

- **Nociceptive pain** is identified using the following criteria^51^:

1. Confirmed proportionate mechanical/anatomical symptom characteristics.
2. Pain comparable to trauma/pathology and in an area of injury or dysfunction with/without referral.
3. Resolution congruent with anticipated tissue healing time.
4. Pain description typically intermittent and sharp with movement/mechanical aggravation.
5. Pain involves additional symptoms of inflammation (e.g., swelling and redness).

- **Central sensitization** is differentiated from neuropathic and nociceptive pain using these criteria^5,51,52^:

When neuropathic pain has been excluded, central sensitization pain is differentiated from nociceptive pain as follows^52^:

1. Pain is out of proportion to the severity of the associated injury or disease.
2. Distribution is diffuse and/or variable, not anatomically congruent with associated injury or disease, with accompanying allodynia or hyperalgesia.
3. Patient is hypersensitive to stimuli such as light, temperature, stress, and emotions.

### Other key terminology and abbreviations

- *Biopsychosocial intervention*: a treatment plan that includes at least one physical component (such as spinal manipulation or exercise) and at least one psychological/social component (such as CBT or mindfulness meditation).^53^
- *CIH*: Complementary and integrative health care.
- *CBT:* Cognitive behavioral therapy, in which unhelpful thought or behavioral patterns are challenged by restructuring thoughts/beliefs and increasing engagement in meaningful activities.
- *MTI:* Maximum Therapeutic Improvement.
- *Psychological and mind–body interventions* focus on interactions among the brain, the rest of the body, the mind, and behavior and the ways in which emotional, mental, social, spiritual, experiential, and behavioral factors affect health. Examples are as follows: psychological therapies such as CBT and mindfulness meditation; physical mind–body therapies such as *t'ai chi*; and yoga.^54^
- *Red flags* are signs or symptoms noted in the history or clinical examination that suggests the possibility of serious pathology or illness requiring immediate referral, more extensive evaluation, or co-management, or present a contraindication to an aspect of the proposed treatment plan.^55,56^
- *Self-care:* An active practice that a person can perform at home independently after being provided with appropriate instruction.^57^
- *SMT:* Spinal manipulative therapy: usually practiced by DC, doctors of osteopathy (DO), or physical therapists (PT).

## Recommendations on Best Practices for Chiropractic Management of Patients with Chronic MSK Pain

### General considerations for chronic pain management

1. *Emphasize the biopsychosocial model*. In keeping with the recommendation of organizations such as the AHRQ and the International Society for the Study of Pain (IASP), management of patients with moderate to severe and/or complicated chronic MSK pain is best addressed within a biopsychosocial model rather than the conventional biomedical model.^6,58^
2. *Prioritize self-management and nonpharmacological approaches.* Self-management and nonpharmacological therapies should be prioritized over pharmacological approaches whenever possible.^3,6–8^
a. For patients on prescribed pain medications, co-management with a provider of nonpharmacological approaches may improve outcomes.^53^
3. *Emphasize active interventions.* Although passive interventions are useful in the initial stages of management to decrease pain, active interventions—particularly exercise and self-care—should be introduced as soon as possible and emphasized in the management plan.^8^
a. Passive interventions, both conventional medical approaches (e.g., medication or surgery) and many nonpharmacological approaches (e.g., acupuncture, massage, spinal manipulation, and physical modalities) should be combined with active interventions and self-care (e.g., exercise, healthy diet,^59^ meditation, yoga, and other lifestyle changes) whenever possible to improve outcomes.^38^
4. *Include both physical and mind–body approaches.* For patients reporting moderate to severe chronic pain, a nonpharmacological approach that includes both a physical and mind–body component is recommended.^53^ These may be administered by the primary treating clinician, or by referral or co-management with an interdisciplinary team.^53^
5. *Identify the neurophysiological type of pain.* In keeping with recent advances in the understanding of the physiology of chronic pain, it is important to differentiate patients' chronic pain in terms of its neurophysiology (neuropathic, nociceptive, and central sensitization), because this may affect treatment choices.^51,60,61^
6. *Consider risk stratification*, such as the STarT Back risk assessment tool, for new episodes of pain to inform shared decisions about treatment approaches. Patients with low risk of a poor outcome may require a less intensive approach, while those with higher risk may require a more intensive approach incorporating multiple therapies, including psychological.^31^

### Informed consent/risks and benefits

1. *Engage the patient in the informed consent process.* Informed consent is a process requiring active communication between the patient and clinician. Using clear and understandable terms, the clinician explains the examination procedures, diagnosis, treatment options (including no treatment), and their benefits and risks.^15^ The clinician should ask the patient if he/she has any questions, and answer them to the patient's satisfaction. The patient must understand this information to make an informed decision.^15^ The informed consent discussion and the patient's consent to proceed should be recorded in the medical record.
2. *Comply with local regulations.* Legal requirements may differ by geographic location; clinicians should seek specific advice from local authorities such as their malpractice carrier or state association. Both the American Chiropractic Association (ACA) and the Association of Chiropractic Colleges (ACC) have guidelines on informed consent.^17,^^†^
3. Maximize patient safety.
a. Nonpharmacological therapies for chronic pain have fewer associated harms than pharmacological interventions, particularly when administered by appropriately trained health professionals.^3^
b. Carefully assess patients with chronic pain for possible contraindications to manipulation, particularly high-velocity, low-amplitude “thrust” maneuvers (Table 9) and red flags (Table 10).^62–64^

### General diagnostic considerations—history, examination, and imaging

#### History and physical examination

1. *Recognize the effect of psychosocial factors on chronic pain physiology.* Chronic pain physiology may be differentiated as nociceptive, neuropathic, and/or central sensitization types. However, pain physiology can manifest in individuals through interactions with psychosocial factors. These may be negative, such as mood or sleep disorders or work-related factors (such as hostile work environment, job insecurity, and long work hours^65,66^) or protective influences such as coping skills and social support.^4,67,68^

**Table 9.** Possible Contraindications to Spinal or Other Joint Manipulation or Mobilization Procedures^15,100^

System	Condition
Musculoskeletal	• Primary or metastatic bone tumors
	• Severe osteoporosis
	• Structural instability (such as unstable spondylolisthesis or postsurgical joint instability)
Inflammatory	• Osteomyelitis
	• Rheumatoid arthritis in the active systemic stage, or locally if acute inflammation or atlantoaxial instability is present
Neurologic	• Progressive or sudden neurologic deficit
	• Spinal cord tumors with neurological compromise or requiring medical intervention
Hematologic	• Any unstable bleeding disorders, including high-dose anticoagulant therapy
	• Unstable aortic aneurysm
Clinician attributes	Inadequate physical examination
	Inadequate manipulative training

Soft-tissue, instrument-assisted manipulation and low-velocity, low-amplitude mobilization procedures may be considered for application, as clinically indicated on an individual basis.^15,100^

**Table 10.** Red Flags on History and Examination^15,17,55,56^

Red flags: History	Red flags: Examination^6^
• Cancer • Confusion/altered consciousness • Connective tissue disease • Osteopenia • Severe nocturnal pain • Significant trauma or infection • Unexplained weight loss • Unexplained/novel neck pain • Visual or speech disturbances • Weakness or loss of sensation • “Worst headache ever” or new headache, unlike any previous	• Abnormal sensory, motor or deep tendon reflexes • Fever >100°F • Nuchal rigidity • Pain pattern unrelated to movements or activities
2. *Take a thorough pain history.* A thorough history of the patient's pain symptoms, previous and concurrent treatment, and psychosocial factors is important to develop an appropriate chiropractic management plan for patients with chronic pain. Components of the history include^17^ the following:
a. Assessment of red and yellow flag risk factors.
b. Onset of current pain and perceptions about initial precipitating factors.
c. Pain parameters, including type, severity, location, frequency, and duration.
d. Provocative and relieving factors.
e. Review of systems.
f. Previous treatment and response, including medical, surgical, and nonpharmacological.
g. History of past, current, or considered self-care strategies.
h. History of diagnostic tests with results.
i . Current medications and nutraceuticals.
j. Complicating factors/barriers to recovery, including social determinants of health^‡^
k. Psychological and behavioral health factors (e.g., depression, stress, anxiety, and PTSD).
l. Lifestyle factors such as tobacco use, drugs/alcohol, diet, exercise, and sedentary lifestyle.
3. *Consider “yellow flags.”* “Yellow Flags” are psychosocial factors that might predict poorer outcomes or prolonged recovery time. They relate to issues such as beliefs about illness and treatment; attitudes and emotional states; and pain behavior.^69^ Examples include^17,69^ the following:
a. Belief that activity should be avoided.
b. Pain catastrophizing.^70^
c. Negative attitude/depression.
d. Work-related stress.
e. Lack of social support.
f. Current compensation and claims issues related to chronic pain.
4. *Consider referral for co-management.* Patients with psychological factors, which may present an obstacle to compliance with or success of the management plan, may benefit by a referral to a psychologist or behavioral health counselor for further evaluation and/or a trial of CBT.^71,72^
5. *Conduct an appropriately focused physical examination.*^73^ Conduct a physical examination informed by symptoms and health history, including areas/sites of primary and secondary symptoms. Both function and pain should be assessed and include a comprehensive MSK and neuromuscular examination.^73^

#### Diagnostic imaging (general considerations and specific recommendations under each condition)

1. *Avoid routine use of imaging.* Because chronic MSK pain is often multifactorial and may not originate from a local source, imaging evidence is rarely capable of definitively identifying a pain source.^73^ However, imaging may be necessary if red flags are present and should be evaluated on a case-by-case basis after a thorough history and examination are performed.

### General treatment considerations

#### Outcome assessment

1. *Use validated Patient-Reported Outcome Measures* to assess patient symptoms and characteristics, and to assess progress over time.^4^ Some Patient-Reported Outcome Measures appropriate for chronic pain chiropractic patients are shown in Table 11.^4,17,74^

**Table 11.** Patient-Reported Outcome Measures for Assessing Chronic Musculoskeletal Pain^4^

Pain characteristics	Functional ability	Quality of life	Psychological factors
Verbal Rating Scale Numeric Rating Scale	Patient Specific Functional Scale (PSFS)^17^	Medical Outcomes Study Short Form Health Survey (SF-36)	Beck Depression Inventory Patient Health Questionnaire (PHQ9)
Visual Analog Scale	Pain Disability Index	Global Well-Being Scale^74^	Profile of Mood States
Neuropathic Pain Scale Central Sensitization Inventory^51,61,101^	Brief Pain Inventory	EuroQol PROMIS Global Health	Coping Strategies Questionnaire PTSD Checklist-Specific Version Alcohol/drug dependency: CAGE-AID^102^

Tools for assessing specific types of pain (low back, etc.) are shown in those sections Only tools for assessing general chronic pain rather than those for specific locations (low back, etc.) are shown.

#### Care pathway

**FIG. 2.**
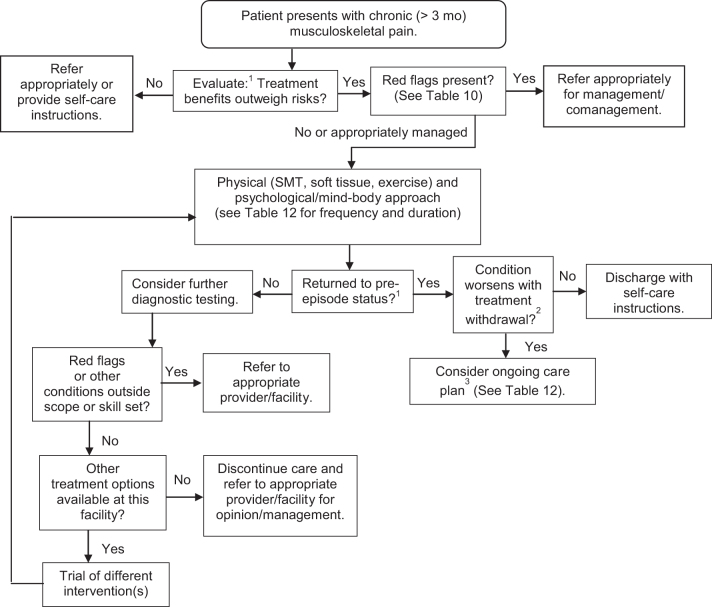
Care pathway for chiropractic management of adult patients with chronic musculoskeletal pain.^14,16^^1^Evaluation and re-evaluation components: History; perform focused examination; imaging if warranted (new trauma/symptoms/red flags); patient-reported outcome measures (PROMs) (Table 11); pain frequency and intensity; functional ability; quality of life; psychological factors. ^2^Attempt treatment withdrawal when patient reaches maximum therapeutic improvement. If improvements deteriorate, ongoing care may be necessary to maintain functional status. Withdrawal can be tapered or abrupt. Either instruct patient to return if symptoms recur; or schedule him/her for re-evaluation at regular intervals. ^3^To document necessity for ongoing care, record: Response to initial treatment (use valid outcome measures); MTI, Maximum Therapeutic Benefit; residual activity limitation; patient's self-care attempts; have alternative treatments been considered or attempted?

1. *Follow an appropriate care pathway.* Figure 2 shows the chiropractic care pathway for a typical adult patient with chronic MSK pain.

#### Considerations for frequency and duration of treatment

1. *Avoid a “curative model” approach.* A “curative model” approach is not likely to be successful with chronic pain management. Pain medications are not expected to “cure” chronic pain, but to make it more manageable for the patient. Similarly, nonpharmacological approaches should not be expected to “cure” chronic pain within a specified course of treatment, but may need to be included as part of an individual's ongoing pain management plan.^15,17,19,36,75,76^ (see Table 12 for details of “Ongoing Management.”)

**Table 12.** Visit Frequency and Duration of Care for Chiropractic Management of Chronic (>3 Months) Musculoskeletal Pain^15,17^

Type of episode	No. of treatment visits	Duration of care	Re-evaluation period
Mild exacerbation	1–6/episode	Per episode	Beginning and end of episode
Moderate or severe exacerbation	2–3/week	2–4 weeks	Every 2–4 weeks
Scheduled interval for ongoing management^36,a,b^	1–4/month	Ongoing	Minimum of every 6 visits, or as needed to document changes.^c^

^a^ Support with documentation of either functional improvement or functional optimization. This may include, but is not limited to the following: (1) substantial symptom recurrence upon treatment withdrawal, (2) minimization/control of pain, (3) maintenance of function and ability to perform, (4) minimization of dependence on interventions with greater risk(s) of adverse events, and (5) maintained or improved work capacity.

^b^ Three to four visits per month on an ongoing basis only indicated in exceptional circumstance. One to two visits per month may be necessary if care is supported by a well-documented care

management plan.

^c^ Document patient's efforts to comply with self-care recommendations.
2. *Set appropriate chronic pain management goals.* The goals of chronic pain management are different from the goals associated with acute care management. Chronic care goals may include (but are not limited to) the following:
a. Pain control: relief to tolerance.
b. Support or maximize patient's current level of function/ADLs.
c. Reduce/minimize reliance on medication.
d. Maximize patient satisfaction.
e. Maximize patient's engagement in meaningful/pleasurable activities to de-emphasize pain (examples: playing with grandchildren; getting hair done; or going to the park)^77,^^#^
f. Minimize exacerbation frequency and/or severity.
g. Minimize further disability.
h. Minimize lost time on the job.
3. *Consider patient-specific goals.* Patients with chronic MSK pain generally fall into one of these categories:
a. *Self-management is sufficient* using strategies/procedures such as exercise, ice, heat, and stress reduction.
b. *Episodic care is necessary to manage pain*. Patients arrange nonpharmacological care on an as-need basis to support their self-care strategies for acute flare-ups, 1–12 visits/episode, followed by release.
c. *Scheduled ongoing physician-directed care is necessary to manage pain.* Treatment withdrawal results in deterioration^36^ (Fig. 2 and Table 12).

### Condition-specific diagnosis and treatment recommendations

This guideline includes recommendations for best practices for chiropractic management of some of the most common chronic MSK pain conditions. These are (1) LBP, (2) neck pain, (3) tension headache, (4) knee and hip OA, and (5) fibromyalgia.^6^

**See General Considerations for Chronic Pain Management section for details of history, examination, and red and yellow flags. Specific considerations for each condition are provided below.**

**1. Chronic LBP**

### Diagnostic considerations for LBP

1. *Develop an evidence-based working diagnosis.* Providers should develop evidence-based working diagnoses that describe condition characteristics that will inform a management approach.^67,68^
2. *Consider physiological pain type.* Providers are advised to consider whether the likely dominant cause of the LBP is neuropathic, nociceptive, and/or due to central sensitization to determine the most appropriate management strategies.^4,5,51,67,68^

### Diagnostic imaging

1. *Avoid routine imaging.* Routine imaging is not recommended for patients with nonspecific LBP.^14,73^ Factors that indicate the need for imaging are^15^ as follows:
a. Severe and/or progressive neurologic deficits.
b. Suspected anatomical anomaly such as spondylolisthesis.
c. Severe trauma.
d. Other red flags on history or physical examination.
e. Patient shows no improvement after a reasonable course of care.
f. Additional factors vary with location and type of pain.
2. *Consider advanced imaging for some cases of radiculopathy.* For patients with CLBP accompanied by radiculopathy, magnetic resonance imaging (MRI) or computed tomography (CT) scans are preferred to plain film radiographs.^15^ Certain conditions that are not detected on physical examination, such as spinal stenosis, may require MRI to be detected.^30^

### Interventions

1. *Consider multiple approaches.* Both active and passive, and both physical and mind–body interventions should be considered in the management plan. The following are recommended, based on current evidence^6,14^
a. Physical active interventions:
Exercise
Yoga/*qigong* (which may also be considered “mind–body” interventions)
Lifestyle advice to stay active; avoid sitting^35^; manage weight if obese^78^; and quit smoking^78,79^
b. Physical passive interventions:
Spinal manipulation/mobilization
Massage
Acupuncture
LLL therapy
Transcutaneous electrical nerve stimulation (TENS) or interferential current may be beneficial as part of a multimodal approach, at the beginning of treatment to assist the patient in becoming or remaining active.^38,47^
c. Combined active and passive: multidisciplinary rehabilitation
d. Psychological/mind–body interventions^80^
CBT
Mindfulness-based stress reduction

**2. Chronic neck pain**

### Diagnostic considerations

*See* General Diagnostic Considerations—History, Examination, and Imaging section

### Diagnostic imaging

*Consider appropriate circumstances for imaging.* According to the American College of Radiology:

1. AP and lateral views of the cervical spine may be appropriate in patients with *a* history of (1) chronic neck pain with or without trauma; (2) malignancy; or (3) neck surgery.^81^
2. Diagnostic imaging to identify degeneration is not recommended because it has not been determined to necessarily be a source of pain.^82^
3. Serial radiographs of the cervical spine are not associated with improved outcomes.^83,84^

### Interventions

1. *Consider multiple approaches.* Both active and passive, and both physical and mind–body interventions should be considered in the management plan for maximum therapeutic effect. The following are recommended, based on current evidence.^6^
a. Physical active interventions^16,18^:
Exercise (range of motion and strengthening).
Exercise combined with manipulation/mobilization.
b. Physical passive interventions:
Spinal manipulation and mobilization^16,18,85^
Massage
Low-level laser
Acupuncture
These modalities may be added as part of a multimodal treatment plan, especially at the beginning, to assist the patient in becoming or remaining active:
Transcutaneous nerve stimulation (TENS), traction, ultrasound, and interferential current.^17,34,37^
c. Mind–body interventions^16,18^
Yoga
qigong

**3. Chronic tension headache**

### Diagnostic considerations for tension headache

By definition, tension-type headache (TTH) is one that is present at least 15 days each month for more than 3 months. It may be daily and unremitting and may be accompanied by mild nausea.^33^ TTH is diagnosed by history exclusively, although a focused examination that includes blood pressure should also be conducted. Imaging and other special tests are not indicated unless the history or examination is suggestive of another condition, which may be the underlying cause.^33^

### Interventions

1. *Consider multiple approaches.* Both active and passive, and both physical and mind–body interventions should be considered in the management plan for maximum therapeutic effect. The following are recommended, based on current evidence^6^:
a. Physical active interventions^33^
Reassurance that TTH does not indicate presence of a disease.
Advice to avoid triggers.
Exercise (aerobic).
b. Physical passive interventions
Spinal manipulation^6,32^
Acupuncture^6,33^
Cold packs or menthol gels^33^
c. Combined active and passive
d. Mind–body interventions^33^
CBT
Relaxation therapy
Biofeedback
Mindfulness Meditation^42^

**4. Knee and hip OA**

### Knee OA

#### Diagnostic considerations for knee OA

1. *Rely first on history and physical examination.* For knee OA, the diagnosis relies on the history and physical examination findings and is often confirmed with plain radiographs. Laboratory tests are reserved to rule out other diagnoses.^86^ It is more common in older adults and in the obese (body mass index >30).^87,88^

#### Diagnostic imaging

1. *Imaging is not typically required.* Imaging is not required for typical presentation of knee OA; however, with chronic knee pain, conventional (plain) radiographs should be utilized before other imaging modalities. Considerations of radiographic views are important for optimizing the detection of knee OA, and specifically, weight bearing and patellofemoral views are recommended.^89,90^
2. *Consider advanced imaging in some cases.* For additional diagnoses, soft tissues are best imaged with diagnostic ultrasound or MRI without contrast, and bone by CT scan or MRI.^89^ Radiographic factors for chronic knee pain in which MRI without IV contrast is usually appropriate to include^89,90^:
a. Negative radiographs
b. Joint effusion
c. Osteochondritis dissecans
d. Loose bodies
e. History of cartilage or meniscal repair
f. Prior osseous injury (i.e., Second fracture and tibial spine avulsion)

### Interventions

1. *Consider multiple approaches.* Both active and passive, and both physical and mind–body interventions should be considered in the management plan. The following are recommended, based on current evidence^6^:
a. Physical active interventions:
Exercise
b. Physical passive interventions:
Manual therapy^39^
Ultrasound
Acupuncture, using “high dose” (greater treatment frequency, at least 3 × week)^41,46^
LLL therapy^45,48^

## Hip OA

### Diagnostic considerations for Hip OA

1. *Develop a clinical diagnosis.* Hip OA commonly presents as anterior or posterior hip pain, with persistent deep groin pain that is worse with activity.^91^ The American College of Rheumatology supports clinical diagnosis of hip OA when patients have hip pain, increased pain on internal hip rotation, and concurrent morning stiffness lasting <60 min.^92^
a. Patients may also have coexisting limitation of flexion with flexion less than or equal to 115° and <15° of internal rotation.^93^

### Diagnostic imaging

1. *First consider plain radiographs.* According to the ACR Appropriateness Criteria for chronic hip pain, the first line of imaging should be plain radiographs of the hip and pelvis for most, if not all, cases. For OA of the hip, physical examination and radiographs may be better for diagnosis than MRI and have reasonable sensitivity and specificity.^92,94^
2. *Consider advanced imaging for signs of cartilage degeneration. MRI* is more sensitive than plain radiographs for detecting early signs of cartilage degeneration. MRI with or without contrast may be indicated if the following are suspected and not confirmed with radiographs^92^:
a. Impingement
b. Labral tears
c. Pigmented villonodular synovitis or osteochromatosis
d. Arthritis of uncertain type
e. Infection

### Interventions

1. *Consider multiple approaches.* Both active and passive, and both physical and mind–body interventions should be considered in the management plan. The following are recommended, based on current evidence^6^
a. Physical active interventions:
Exercise
b. Physical passive interventions
Manual therapy^40^

**5. Fibromyalgia**

### Diagnostic considerations for fibromyalgia

Fibromyalgia is diagnosed primarily from a history of a typical cluster of symptoms—widespread chronic pain, nonrestorative sleep, and fatigue (physical and/or mental)—when other possible causes have been excluded.^95^

### Interventions

1. *Consider multiple approaches.* Both active and passive, and both physical and mind–body interventions should be considered in the management plan. The following are recommended, based on current evidence^6,95,96^:
a. Physical active interventions:
Exercise (aerobic and strengthening)
Advice on healthy lifestyle^95^
Education on the condition^95^
b. Physical passive interventions:
Spinal manipulation^97^
Myofascial release^97^
Acupuncture^44^
LLL therapy^43,49^
c. Combined active and passive: multidisciplinary rehabilitation
d. Mind–body interventions, including CBT, mindfulness meditation, yoga, and *t'ai chi*, *qigong*

## Discussion

The management of chronic pain has seen a dramatic shift recently, with nonpharmacological approaches being preferred to pharmacological, due to the opioid epidemic. Therefore, the management of chronic pain patients is not the domain of any one type of provider. In addition, evidence supports the biopsychosocial approach that includes not only multifactorial treatment approach but also a strong emphasis on psychosocial factors, active care, self-care, and patient empowerment.

This guideline is meant to emphasize the use of evidence-based approaches to chronic MSK pain management that help patients become active as soon as possible and empower themselves to manage their pain successfully. It also aims to encourage DCs to work collaboratively with other providers to provide patients with the optimal resources for successfully managing their chronic pain.

A limitation in making such recommendations is that some treatment practices in common use may not have accumulated the highest quality evidence. However, it is important to give practitioners as much guidance as possible, using the best *available* evidence, as Sackett first described it.^98^

There are factors that contribute to the relative scarcity of high-quality evidence for nonpharmacological treatments, particularly manual therapies, for chronic pain. One is that randomized controlled trials of nonpharmacological treatments, particularly manual therapies, usually assume a curative model.^75^ For example, RCTs usually test the hypothesis that a course of spinal manipulative therapy (SMT) will result in long-term pain reduction—a curative model—and if it does not, then SMT is considered ineffective.^75^ However, chronic MSK pain is not medically managed in that same curative model. Analgesics are not expected to function like antibiotics—that is, to “cure” pain after a course of treatment. Although some studies are beginning to approach the topic of chronic pain from a management, rather than curative, approach,^36,75,99^ currently, the literature is still scarce on optimal treatment parameters, and future studies are important to conduct.

After our project was completed and we were preparing this article, AHRQ published a 2020 update^9^ to their 2018 review,^6^ which had formed the foundation of our recommendations. We found that their 2020 update did not substantively alter our recommendations. The fact that AHRQ saw fit to produce an update so quickly emphasizes the importance of the topic of nonpharmacological approaches to chronic MSK pain.

We sought to secure buy-in from the chiropractic profession in developing this guideline by forming a large and broad-based Delphi panel and by disseminating the preliminary recommendations very widely throughout the profession. We hope that the consensus achieved will facilitate their use in chiropractic practice. We also hope that these evidence-based recommendations for a variety of conservative treatment approaches to the management of common chronic MSK pain conditions will foster collaboration between provider groups, and thereby improve patient outcomes.

## Supplementary Material

Supplemental data

Supplemental data

## Notes

^*^ www.essentialevidenceplus.com/product/ebm_loe.cfm?show=grade.

^†^ www.chirocolleges.org/resources/informed-consent-guideline

^‡^ https://www.healthypeople.gov/2020/topics-objectives/topic/social-determinants-of-health

^#^ https://www.va.gov/PAINMANAGEMENT/docs/CBT-CP_Therapist_Manual.pdf

## References

[1] Nahin RL, Sayer B, Stussman BJ, Feinberg TM Eighteen-year trends in the prevalence of, and health care use for, noncancer pain in the United States: Data from the Medical Expenditure Panel Survey. J Pain 2019;20:796–809 30658177 10.1016/j.jpain.2019.01.003

[2] Dahlhamer J, Lucas J, Zelaya C, et al. Prevalence of chronic pain and high-impact chronic pain among adults—United States, 2016. MMWR Morb Mortal Wkly Rep 2018;67:1001–1006 30212442 10.15585/mmwr.mm6736a2 PMC6146950

[3] Qaseem A, Wilt TJ, McLean RM, et al. Noninvasive treatments for acute, subacute, and chronic low back pain: A clinical practice guideline from the American College of Physicians. Ann Intern Med 2017;166:514–530 28192789 10.7326/M16-2367

[4] Clauw DJ, Essex MN, Pitman V, Jones KD Reframing chronic pain as a disease, not a symptom: Rationale and implications for pain management. Postgrad Med 2019;131:185–198 30700198 10.1080/00325481.2019.1574403

[5] Treede RD, Rief W, Barke A, et al. A classification of chronic pain for ICD-11. Pain 2015;156:1003–1007 25844555 10.1097/j.pain.0000000000000160 PMC4450869

[6] Skelly AC, Chou R, Dettori JR, et al. Noninvasive Nonpharmacological Treatment for Chronic Pain: A Systematic Review. Rockland, MD: AHRQ, 2018 30179389

[7] Institute of Medicine. Relieving Pain in America. Washington, DC: IOM, 2011

[8] National Pain Strategy Task Force. National Pain Strategy: A Comprehensive Population Health-Level Strategy for Pain. Bethesda, MD: National Institutes of Health, 2015

[9] Agency for Healthcare Research and Quality. Noninvasive Nonpharmacological Treatment for Chronic Pain: A Systematic Review Update. Rockville, MD: U.S. Department of Health and Human Services, 2020 32338846

[10] Lemmon R, Hampton A Nonpharmacologic treatment of chronic pain: What works? J Fam Pract 2018;67:474;477;480;483. 30110500

[11] National Board of Chiropractic Examiners. Practice Analysis of Chiropractic, 2020. Greeley, CO: National Board of Chiropractic Examiners, 2020

[12] Tick H, Nielsen A, Pelletier KR, et al. Evidence-based nonpharmacologic strategies for comprehensive pain care: The Consortium Pain Task Force White Paper. Explore (NY) 2018;14:177–211 29735382 10.1016/j.explore.2018.02.001

[13] Timmons E, Hockenberry JM, Durrance CP More battles among licensed occupations: Estimating the effects of scope of practice and direct access on the chiropractic, physical therapist, and physician labor market. Mercatus Res 2016:1–29

[14] Bussieres AE, Stewart G, Al-Zoubi F, et al. Spinal manipulative therapy and other conservative treatments for low back pain: A guideline From the Canadian Chiropractic Guideline Initiative. J Manipulative Physiol Ther 2018;41:265–293 29606335 10.1016/j.jmpt.2017.12.004

[15] Globe G, Farabaugh RJ, Hawk C, et al. Clinical practice guideline: Chiropractic care for low back pain. J Manipulative Physiol Ther 2016;39:1–22 26804581 10.1016/j.jmpt.2015.10.006

[16] Bussieres AE, Stewart G, Al-Zoubi F, et al. The treatment of neck pain-associated disorders and whiplash-associated disorders: A clinical practice guideline. J Manipulative Physiol Ther 2016;39:523–564 e527. 27836071 10.1016/j.jmpt.2016.08.007

[17] Whalen W, Farabaugh RJ, Hawk C, et al. Best-practice recommendations for chiropractic management of patients with neck pain. J Manipulative Physiol Ther 2019;42:635–650 31870638 10.1016/j.jmpt.2019.08.001

[18] Cote P, Wong JJ, Sutton D, et al. Management of neck pain and associated disorders: A clinical practice guideline from the Ontario Protocol for Traffic Injury Management (OPTIMa) Collaboration. Eur Spine J 2016;25:2000–2022 26984876 10.1007/s00586-016-4467-7

[19] Farabaugh RJ, Dehen MD, Hawk C Management of chronic spine-related conditions: Consensus recommendations of a multidisciplinary panel. J Manipulative Physiol Ther 2010;33:484–492 20937426 10.1016/j.jmpt.2010.07.002

[20] Vernooij RW, Sanabria AJ, Sola I, et al. Guidance for updating clinical practice guidelines: A systematic review of methodological handbooks. Implement Sci 2014;9:3 24383701 10.1186/1748-5908-9-3 PMC3904688

[21] Brouwers M, Kho M, Browman GP, et al. Advancing guideline development, reporting and evaluation in healthcare. Can Med Assoc J 2010;182:E839–E842 20603348 10.1503/cmaj.090449 PMC3001530

[22] Harbour R, Lowe G, Twaddle S Scottish Intercollegiate Guidelines Network: The first 15 years (1993–2008). J R Coll Physicians Edinb 2011;41:163–168 21677923 10.4997/JRCPE.2011.209

[23] Hawk C, Minkalis AL, Khorsan R, et al. Systematic review of nondrug, nonsurgical treatment of shoulder conditions. J Manipulative Physiol Ther 2017;40:293–319 28554433 10.1016/j.jmpt.2017.04.001

[24] Hawk C, Minkalis A, Webb C, et al. Manual interventions for musculoskeletal factors in infants with suboptimal breastfeeding: A scoping review. Evid Based Integr Med 2018;23:1–12

[25] Guyatt GH, Oxman AD, Vist GE, et al. GRADE: An emerging consensus on rating quality of evidence and strength of recommendations. BMJ 2008;336:924–926 18436948 10.1136/bmj.39489.470347.AD PMC2335261

[26] Fitch K, Bernstein S, Aquilar MS, et al. The RAND UCLA Appropriateness Method User's Manual. Santa Monica, CA: RAND Corporation; 2003

[27] Luque-Suarez A, Martinez-Calderon J, Falla D Role of kinesiophobia on pain, disability and quality of life in people suffering from chronic musculoskeletal pain: A systematic review. Br J Sports Med 2019;53:554–559 29666064 10.1136/bjsports-2017-098673

[28] Lin I, Wiles LK, Waller R, et al. Poor overall quality of clinical practice guidelines for musculoskeletal pain: A systematic review. Br J Sports Med 2018;52:337–343 29175827 10.1136/bjsports-2017-098375

[29] Bier JD, Scholten-Peeters WGM, Staal JB, et al. Clinical practice guideline for physical therapy assessment and treatment in patients with nonspecific neck pain. Phys Ther 2018;98:162–171 29228289 10.1093/ptj/pzx118

[30] Blanpied PR, Gross AR, Elliott JM, et al. Neck pain: Revision 2017. J Orthop Sports Phys Ther 2017;47:A1–A83 10.2519/jospt.2017.0302 28666405

[31] National Guideline Center. Low Back Pain and Sciatica in Over 16s: Assessment and Management. London: National Institute for Health and Care Excellence, 2016. 27929617

[32] Cote P, Yu H, Shearer HM, et al. Non-pharmacological management of persistent headaches associated with neck pain: A clinical practice guideline from the Ontario protocol for traffic injury management (OPTIMa) collaboration. Eur J Pain 2019;23:1051–1070 30707486 10.1002/ejp.1374

[33] Steiner TJ, Jensen R, Katsarava Z, et al. Aids to management of headache disorders in primary care (2nd edition): On behalf of the European Headache Federation and Lifting The Burden: The Global Campaign against Headache. J Headache Pain 2019;20:57 31113373 10.1186/s10194-018-0899-2 PMC6734476

[34] Albornoz-Cabello M, Perez-Marmol JM, Barrios Quinta CJ, et al. Effect of adding interferential current stimulation to exercise on outcomes in primary care patients with chronic neck pain: A randomized controlled trial. Clin Rehabil 2019;33:1458–1467 31007047 10.1177/0269215519844554

[35] Barone Gibbs B, Hergenroeder AL, Perdomo SJ, et al. Reducing sedentary behaviour to decrease chronic low back pain: The stand back randomised trial. Occup Environ Med 2018;75:321–327 29330230 10.1136/oemed-2017-104732 PMC8283944

[36] Eklund A, Jensen I, Lohela-Karlsson M, et al. The Nordic Maintenance Care program: Effectiveness of chiropractic maintenance care versus symptom-guided treatment for recurrent and persistent low back pain-A pragmatic randomized controlled trial. PLoS One 2018;13:e0203029 30208070 10.1371/journal.pone.0203029 PMC6135505

[37] Yesil H, Hepguler S, Dundar U, et al. Does the use of electrotherapies increase the effectiveness of neck stabilization exercises for improving pain, disability, mood, and quality of life in chronic neck pain? A Randomized, Controlled, Single Blind Study. Spine (Phila Pa 1976) 2018;43:E1174–E1183 29652778 10.1097/BRS.0000000000002663

[38] Almeida CC, Silva V, Junior GC, et al. Transcutaneous electrical nerve stimulation and interferential current demonstrate similar effects in relieving acute and chronic pain: A systematic review with meta-analysis. Braz J Phys Ther 2018;22:347–354 29426587 10.1016/j.bjpt.2017.12.005 PMC6157468

[39] Anwer S, Alghadir A, Zafar H, Brismee JM Effects of orthopaedic manual therapy in knee osteoarthritis: A systematic review and meta-analysis. Physiotherapy 2018;104:264–276 30030035 10.1016/j.physio.2018.05.003

[40] Ceballos-Laita L, Estebanez-de-Miguel E, Martin-Nieto G, et al. Effects of non-pharmacological conservative treatment on pain, range of motion and physical function in patients with mild to moderate hip osteoarthritis. A systematic review. Complement Ther Med 2019;42:214–222 30670244 10.1016/j.ctim.2018.11.021

[41] Gong Z, Liu R, Yu W, et al. Acutherapy for knee osteoarthritis relief in the elderly: A systematic review and meta-analysis. Evid Based Complement Alternat Med 2019;2019:1868107 30906410 10.1155/2019/1868107 PMC6398067

[42] Gu Q, Hou JC, Fang XM Mindfulness meditation for primary headache pain: A meta-analysis. Chin Med J (Engl) 2018;131:829–838 29578127 10.4103/0366-6999.228242 PMC5887742

[43] Honda Y, Sakamoto J, Hamaue Y, et al. Effects of physical-agent pain relief modalities for fibromyalgia patients: A systematic review and meta-analysis of randomized controlled trials. Pain Res Manag 2018;2018:2930632 30402199 10.1155/2018/2930632 PMC6191958

[44] Kim J, Kim SR, Lee H, Nam DH Comparing verum and sham acupuncture in fibromyalgia syndrome: A systematic review and meta-analysis. Evid Based Complement Alternat Med 2019;2019:8757685 31534469 10.1155/2019/8757685 PMC6732586

[45] Stausholm MB, Naterstad IFM, Joensen J, et al. Efficacy of low-level laser therapy on pain and disability in knee osteoarthritis: Systematic review and meta-analysis of randomised placebo-controlled trials. BMJ Open 2019;9:e031142 10.1136/bmjopen-2019-031142 PMC6830679 31662383

[46] Sun N, Tu JF, Lin LL, et al. Correlation between acupuncture dose and effectiveness in the treatment of knee osteoarthritis: A systematic review. Acupunct Med 2019;37:261–267 31271300 10.1136/acupmed-2017-011608

[47] Wu LC, Weng PW, Chen CH, et al. Literature review and meta-analysis of transcutaneous electrical nerve stimulation in treating chronic back pain. Reg Anesth Pain Med 2018;43:425–433 29394211 10.1097/AAP.0000000000000740 PMC5916478

[48] Wyszynska J, Bal-Bochenska M Efficacy of high-intensity laser therapy in treating knee osteoarthritis: A first systematic review. Photomed Laser Surg 2018;36:343–353 29688827 10.1089/pho.2017.4425

[49] Yeh SW, Hong CH, Shih MC, et al. Low-level laser therapy for fibromyalgia: A systematic review and meta-analysis. Pain Physician 2019;22:241–254 31151332

[50] Treede RD, Jensen TS, Campbell JN, et al. Neuropathic pain: Redefinition and a grading system for clinical and research purposes. Neurology 2008;70:1630–1635 18003941 10.1212/01.wnl.0000282763.29778.59

[51] Nijs J, Apeldoorn A, Hallegraeff H, et al. Low back pain: Guidelines for the clinical classification of predominant neuropathic, nociceptive, or central sensitization pain. Pain Physician 2015;18:E333–E346 26000680

[52] Nijs J, Torres-Cueco R, van Wilgen CP, et al. Applying modern pain neuroscience in clinical practice: Criteria for the classification of central sensitization pain. Pain Physician 2014;17:447–457 25247901

[53] Kamper SJ, Apeldoorn AT, Chiarotto A, et al. Multidisciplinary biopsychosocial rehabilitation for chronic low back pain: Cochrane systematic review and meta-analysis. BMJ 2015;350:h444 25694111 10.1136/bmj.h444 PMC4353283

[54] Health NCfCaI. Mind and Body Approaches for Chronic Pain: What the Science Says. Bethesda, MD: National Institutes of Health, 2019

[55] Vijiaratnam N, Williams DR, Bertram KL Neck pain: What if it is not musculoskeletal? Aust J Gen Pract 2018;47:279–282 29779295 10.31128/AFP-10-17-4358

[56] Cohen SP, Hooten WM Advances in the diagnosis and management of neck pain. BMJ 2017;358:j3221 28807894 10.1136/bmj.j3221

[57] Crawford C, Lee C, Buckenmaier C III, et al. The current state of the science for active self-care complementary and integrative medicine therapies in the management of chronic pain symptoms: Lessons learned, directions for the future. Pain Med 2014;15 Suppl 1:S104–S113 24734856 10.1111/pme.12406

[58] International Society for the Study of Pain. Evidence-Based Biopsychosocial Treatment of Chronic Musculoskeletal Pain. Washington DC: International Association for Pain, 2017

[59] Rondanelli M, Faliva MA, Miccono A, et al. Food pyramid for subjects with chronic pain: Foods and dietary constituents as anti-inflammatory and antioxidant agents. Nutr Res Rev 2018;31:131–151 29679994 10.1017/S0954422417000270

[60] Nijs J, Clark J, Malfliet A, et al. In the spine or in the brain? Recent advances in pain neuroscience applied in the intervention for low back pain. Clin Exp Rheumatol 2017;35 Suppl 107:108–115 28967357

[61] Nijs J, Polli A, Willaert W, et al. Central sensitisation: Another label or useful diagnosis? Drug Ther Bull 2019;57:60–63 30858291 10.1136/dtb.2018.000035

[62] Herman PM, Vernon H, Hurwitz EL, et al. Clinical scenarios for which cervical mobilization and manipulation are considered by an expert panel to be appropriate (and inappropriate) for patients with chronic neck pain. Clin J Pain 2020;36:273–280 31985500 10.1097/AJP.0000000000000800 PMC7071980

[63] Whedon JM, Mackenzie TA, Phillips RB, Lurie JD Risk of traumatic injury associated with chiropractic spinal manipulation in Medicare Part B beneficiaries aged 66 to 99 years. Spine (Phila Pa 1976) 2015;40:264–270 25494315 10.1097/BRS.0000000000000725 PMC4326543

[64] Whedon JM, Song Y, Mackenzie TA, et al. Risk of stroke after chiropractic spinal manipulation in medicare B beneficiaries aged 66 to 99 years with neck pain. J Manipulative Physiol Ther 2015;38:93–101 25596875 10.1016/j.jmpt.2014.12.001 PMC4336806

[65] Yang H, Haldeman S, Lu ML, Baker D Low back pain prevalence and related workplace psychosocial risk factors: A study using data from the 2010 National Health Interview Survey. J Manipulative Physiol Ther 2016;39:459–472 27568831 10.1016/j.jmpt.2016.07.004 PMC5530370

[66] Yang H, Hitchcock E, Haldeman S, et al. Workplace psychosocial and organizational factors for neck pain in workers in the United States. Am J Ind Med 2016;59:549–560 27184340 10.1002/ajim.22602 PMC4979741

[67] Vining RD, Minkalis AL, Shannon ZK, Twist EJ Development of an Evidence-Based Practical Diagnostic Checklist and corresponding clinical exam for low back pain. J Manipulative Physiol Ther 2019;42:665–676 31864770 10.1016/j.jmpt.2019.08.003

[68] Vining RD, Shannon ZK, Minkalis AL, Twist EJ Current evidence for diagnosis of common conditions causing low back pain: Systematic review and standardized terminology recommendations. J Manipulative Physiol Ther 2019;42:651–664 31870637 10.1016/j.jmpt.2019.08.002

[69] Nicholas MK, Linton SJ, Watson PJ, et al. Early identification and management of psychological risk factors (“yellow flags”) in patients with low back pain: A reappraisal. Phys Ther 2011;91:737–753 21451099 10.2522/ptj.20100224

[70] Martinez-Calderon J, Jensen MP, Morales-Asencio JM, Luque-Suarez A Pain catastrophizing and function in individuals with chronic musculoskeletal pain: A systematic review and meta-analysis. Clin J Pain 2019;35:279–293 30664551 10.1097/AJP.0000000000000676

[71] Monticone M, Ambrosini E, Vernon H, et al. Efficacy of two brief cognitive-behavioral rehabilitation programs for chronic neck pain: Results of a randomized controlled pilot study. Eur J Phys Rehabil Med 2018;54:890–899 29984567 10.23736/S1973-9087.18.05206-1

[72] Monticone M, Vernon H, Brunati R, et al. The NeckPix((c)): Development of an evaluation tool for assessing kinesiophobia in subjects with chronic neck pain. Eur Spine J 2015;24:72–79 25115918 10.1007/s00586-014-3509-2

[73] Carlson H, Carlson N An overview of the management of persistent musculoskeletal pain. Ther Adv Musculoskelet Dis 2011;3:91–99 22870469 10.1177/1759720X11398742 PMC3382682

[74] Khorsan R, Coulter ID, Hawk C, Choate CG Measures in chiropractic research: Choosing patient-based outcome assessments. J Manipulative Physiol Ther 2008;31:355–375 18558278 10.1016/j.jmpt.2008.04.007

[75] Herman P, Edgington S, Ryan G, Coulter I Prevalence and characteristics of chronic spinal pain patients with different hopes (treatment goals) for ongoing chiropractic care. J Altern Complement Med 2019;25:1015–1025 31453711 10.1089/acm.2019.0247 PMC6802729

[76] Dehen MD, Whalen WM, Farabaugh RJ, Hawk C Consensus terminology for stages of care: Acute, chronic, recurrent, and wellness. J Manipulative Physiol Ther 2010;33:458–463 20732583 10.1016/j.jmpt.2010.06.007

[77] Murphy J, McKellar JD, Raffa SD, et al. Cognitive Behavioral Therapy for Chronic Pain among Veterans: Therapist Manual. Washington, DC: US Department of Veterans Affairs, 2014

[78] Suri P, Boyko EJ, Smith NL, et al. Modifiable risk factors for chronic back pain: Insights using the co-twin control design. Spine J 2017;17:4–14 27794503 10.1016/j.spinee.2016.07.533 PMC6126929

[79] Petre B, Torbey S, Griffith JW, et al. Smoking increases risk of pain chronification through shared corticostriatal circuitry. Hum Brain Mapp 2015;36:683–694 25307796 10.1002/hbm.22656 PMC4893785

[80] Institute for Clinical and Economic Review. Cognitive and Mind-Body Therapies for Chronic Low Back and Neck Pain: Effectiveness and Value. Institute for Clinical and Economic Review, 2017

[81] American College of Radiology American College of Radiology ACR Appropriateness Criteria for Chronic Neck Pain. Reston, VA: American College of Radiology, 2013 11037447

[82] Brinjikji W, Luetmer PH, Comstock B, et al. Systematic literature review of imaging features of spinal degeneration in asymptomatic populations. AJNR Am J Neuroradiol 2015;36:811–816 25430861 10.3174/ajnr.A4173 PMC4464797

[83] Bussieres AE, Peterson C, Taylor JA Diagnostic imaging guideline for musculoskeletal complaints in adults-an evidence-based approach-part 2: Upper extremity disorders. J Manipulative Physiol Ther 2008;31:2–32 18308152 10.1016/j.jmpt.2007.11.002

[84] Triano JJ, Budgell B, Bagnulo A, et al. Review of methods used by chiropractors to determine the site for applying manipulation. Chiropr Man Therap 2013;21:36 10.1186/2045-709X-21-36 PMC4028787 24499598

[85] Coulter ID, Crawford C, Vernon H, et al. Manipulation and mobilization for treating chronic nonspecific neck pain: A systematic review and meta-analysis for an Appropriateness Panel. Pain Physician 2019;22:E55–E70 30921975 PMC6800035

[86] Lespasio MJ, Piuzzi NS, Husni ME, et al. Knee osteoarthritis: A primer. Perm J 2017;21:16–183 10.7812/TPP/16-183 PMC5638628 29035179

[87] Arden N, Nevitt MC Osteoarthritis: Epidemiology. Best Pract Res Clin Rheumatol 2006;20:3–25 16483904 10.1016/j.berh.2005.09.007

[88] Kulkarni K, Karssiens T, Kumar V, Pandit H Obesity and osteoarthritis. Maturitas 2016;89:22–28 27180156 10.1016/j.maturitas.2016.04.006

[89] Sakellariou G, Conaghan PG, Zhang W, et al. EULAR recommendations for the use of imaging in the clinical management of peripheral joint osteoarthritis. Ann Rheum Dis 2017;76:1484–1494 28389554 10.1136/annrheumdis-2016-210815

[90] Expert Panel on Musculoskeletal I; Fox MG, Chang EY, et al. ACR Appropriateness Criteria((R)) Chronic Knee Pain. J Am Coll Radiol 2018;15(11S):S302–S312 30392599 10.1016/j.jacr.2018.09.016

[91] Battaglia PJ, D'Angelo K, Kettner NW Posterior, lateral, and anterior hip pain due to musculoskeletal origin: A narrative literature review of history, physical examination, and diagnostic imaging. J Chiropr Med 2016;15:281–293 27857636 10.1016/j.jcm.2016.08.004 PMC5106442

[92] Expert Panel on Musculoskeletal I, Mintz DN, Roberts CC, et al. ACR Appropriateness Criteria((R)) Chronic Hip Pain. J Am Coll Radiol 2017;14(5S):S90–S102 28473098 10.1016/j.jacr.2017.01.035

[93] Altman R, Alarcon G, Appelrouth D, et al. The American College of Rheumatology criteria for the classification and reporting of osteoarthritis of the hip. Arthritis Rheum 1991;34:505–514 2025304 10.1002/art.1780340502

[94] Xu L, Hayashi D, Guermazi A, et al. The diagnostic performance of radiography for detection of osteoarthritis-associated features compared with MRI in hip joints with chronic pain. Skeletal Radiol 2013;42:1421–1428 23842574 10.1007/s00256-013-1675-7

[95] Hauser W, Ablin J, Perrot S, Fitzcharles MA Management of fibromyalgia: Practical guides from recent evidence-based guidelines. Pol Arch Intern Med 2017;127:47–56 28075425 10.20452/pamw.3877

[96] Lauche R, Cramer H, Hauser W, et al. A systematic overview of reviews for complementary and alternative therapies in the treatment of the fibromyalgia syndrome. Evid Based Complement Alternat Med 2015;2015:610615 26246841 10.1155/2015/610615 PMC4515506

[97] Clar C, Tsertsvadze A, Court R, et al. Clinical effectiveness of manual therapy for the management of musculoskeletal and non-musculoskeletal conditions: Systematic review and update of UK evidence report. Chiropr Man Therap 2014;22:12 10.1186/2045-709X-22-12 PMC3997823 24679336

[98] Sackett DL, Rosenberg WM, Gray JA, et al. Evidence based medicine: What it is and what it isn't. BMJ 1996;312:71–72 8555924 10.1136/bmj.312.7023.71 PMC2349778

[99] Eklund A, Hagberg J, Jensen I, et al. The Nordic maintenance care program: Maintenance care reduces the number of days with pain in acute episodes and increases the length of pain free periods for dysfunctional patients with recurrent and persistent low back pain—A secondary analysis of a pragmatic randomized controlled trial. Chiropr Man Ther 2020;28:19 10.1186/s12998-020-00309-6 PMC7171853 32316995

[100] Todd AJ, Carroll MT, Mitchell EKL Forces of commonly used chiropractic techniques for children: A review of the literature. J Manipulative Physiol Ther 2016;39:401–410 27346861 10.1016/j.jmpt.2016.05.006

[101] Scerbo T, Colasurdo J, Dunn S, et al. Measurement properties of the Central Sensitization Inventory: A systematic review. Pain Pract 2018;18:544–554 28851012 10.1111/papr.12636

[102] Brown RL, Rounds LA Conjoint screening questionnaires for alcohol and other drug abuse: Criterion validity in a primary care practice. Wis Med J 1995;94:135–140 7778330

[103] Grading of Recommendations Assessment, Development, and Evaluation. Working Group 2007 1 (modified by the EBM Guidelines Editorial Team). Online document at: www.essentialevidenceplus.com/product/ebm_loe.cfm?show=grade, accessed August 11, 2019

