# Supplementary material for: Best Practices for Chiropractic Management of Patients with Chronic Musculoskeletal Pain: A Clinical Practice Guideline
Source: J Altern Complement Med. 2020 Oct 8;26(10):884–901. doi: 10.1089/acm.2020.0181 (PMC7578188; doi:10.1089/acm.2020.0181)
Supplement: Supplemental data [file Supp_Data1.docx]

**Search Strategy**

**PubMed search string**

(noninvasive or non-invasive or non-pharmacologic* or nonpharmacologic* or "manual therapy" or "manipulation, chiropractic" or "chiropractic" or "musculoskeletal manipulations" or "manipulation, spinal" or spinal manipulation or “manipulation, osteopathic” or osteopathic manipulation or alternative medicine or complementary therapies or acupuncture or biofeedback or mindfulness or psychotherapy or cognitive behavioral therapy or yoga or tai chi or qigong or Pilates or “mind-body” or relaxation or massage or exercise or traction or “ultrasound” or transcutaneous electrical nerve stimulation or low-level laser therapy or interferential or “superficial heat” or “superficial cold” or bracing or physical therapy or stimulation or craniosacral or meditation or “functional restoration” or multidiscipline* or interdisciplin*)) AND (((osteoarthritis or fibromyalgia)) OR (((back or lumbar or lumbo* or spine or spinal or neck or cervical or whiplash or temporomandibular or radicular or radiculopathy or headache)) AND (pain and chronic))) Filters: Guideline; Meta-Analysis; Systematic Reviews; Publication date from 2017/01/01; Humans; English

Cochrane Database of Systematic Reviews – 14 results 8.15.19

| 1 | back or lumbar or lumbo* or spine or spinal or neck or cervical or whiplash or temporomandibular or radicular or radiculopathy or headache | 894 |
| --- | --- | --- |
| 2 | pain and chronic | 463 |
| 3 | 1 and 2 | 147 |
| 4 | osteoarthritis or fibromyalgia | 162 |
| 5 | 3 or 4 | 295 |
| 6 | noninvasive or non-invasive or non-pharmacologic* or nonpharmacologic* or "manual therapy" or "manipulation, chiropractic" or "chiropractic" or "musculoskeletal manipulations" or "manipulation, spinal" or spinal manipulation or “manipulation, osteopathic” or osteopathic manipulation or alternative medicine or complementary therapies or acupuncture or biofeedback or mindfulness or psychotherapy or cognitive behavioral therapy or yoga or tai chi or qigong or Pilates or “mind-body” or relaxation or massage or exercise or traction or “ultrasound” or transcutaneous electrical nerve stimulation or low-level laser therapy or interferential or “superficial heat” or “superficial cold” or bracing or physical therapy or stimulation or craniosacral or meditation or “functional restoration” or multidiscipline* or interdisciplin* | 1719 |
| 7 | 5 and 6 | 125 |
| 8 | Limit 2017 - 2019 | 14 |

PubMed/MEDLINE – 327 results 8.15.19

| 1 | back or lumbar or lumbo* or spine or spinal or neck or cervical or whiplash or temporomandibular or radicular or radiculopathy or headache | 1249312 |
| --- | --- | --- |
| 2 | pain and chronic | 117914 |
| 3 | 1 and 2 | 37859 |
| 4 | osteoarthritis or fibromyalgia | 94557 |
| 5 | 3 or 4 | 130418 |
| 6 | noninvasive or non-invasive or non-pharmacologic* or nonpharmacologic* or "manual therapy" or "manipulation, chiropractic" or "chiropractic" or "musculoskeletal manipulations" or "manipulation, spinal" or spinal manipulation or “manipulation, osteopathic” or osteopathic manipulation or alternative medicine or complementary therapies or acupuncture or biofeedback or mindfulness or psychotherapy or cognitive behavioral therapy or yoga or tai chi or qigong or Pilates or “mind-body” or relaxation or massage or exercise or traction or “ultrasound” or transcutaneous electrical nerve stimulation or low-level laser therapy or interferential or “superficial heat” or “superficial cold” or bracing or physical therapy or stimulation or craniosacral or meditation or “functional restoration” or multidiscipline* or interdisciplin* | 2359968 |
| 7 | 5 and 6 | 30309 |
| 8 | Limit 01/01/2017-08/15/19 | 6478 |
| 9 | Limit 8 to meta-analysis or systematic reviews or guideline | 461 |
| 10 | Limit 9 to English language | 447 |
| 11 | Limit 10 to Humans | 327 |
