# Supplementary material for: Best Practices for Chiropractic Management of Patients with Chronic Musculoskeletal Pain: A Clinical Practice Guideline
Source: J Altern Complement Med. 2020 Oct 8;26(10):884–901. doi: 10.1089/acm.2020.0181 (PMC7578188; doi:10.1089/acm.2020.0181)
Supplement: Supplemental data [file Supp_Data2.docx]

**Supplementary Materials: Excluded Studies**

1. Afzali T, Fangel MV, Vestergaard AS, Rathleff MS, Ehlers LH, Jensen MB. Cost-effectiveness of treatments for non-osteoarthritic knee pain conditions: A systematic review. *PloS one.* 2018;13(12):e0209240.

2. Ahern M, Skyllas J, Wajon A, Hush J. The effectiveness of physical therapies for patients with base of thumb osteoarthritis: Systematic review and meta-analysis. *Musculoskeletal science & practice.* 2018;35:46-54.

3. Aiyer R, Barkin RL, Bhatia A, Gungor S. A systematic review on the treatment of phantom limb pain with spinal cord stimulation. *Pain management.* 2017;7(1):59-69.

4. Al Bimani SA, Gates LS, Warner M, Bowen C. Factors influencing return to play following conservatively treated ankle sprain: a systematic review. *The Physician and sportsmedicine.* 2019;47(1):31-46.

5. Al Zoubi FM, Menon A, Mayo NE, Bussieres AE. The effectiveness of interventions designed to increase the uptake of clinical practice guidelines and best practices among musculoskeletal professionals: a systematic review. *BMC health services research.* 2018;18(1):435.

6. Alanazi MH, Parent EC, Dennett E. Effect of stabilization exercise on back pain, disability and quality of life in adults with scoliosis: a systematic review. *European journal of physical and rehabilitation medicine.* 2018;54(5):647-653.

7. Alentorn-Geli E, Samuelsson K, Musahl V, Green CL, Bhandari M, Karlsson J. The Association of Recreational and Competitive Running With Hip and Knee Osteoarthritis: A Systematic Review and Meta-analysis. *The Journal of orthopaedic and sports physical therapy.* 2017;47(6):373-390.

8. Alrushud AS, Rushton AB, Kanavaki AM, Greig CA. Effect of physical activity and dietary restriction interventions on weight loss and the musculoskeletal function of overweight and obese older adults with knee osteoarthritis: a systematic review and mixed method data synthesis. *BMJ open.* 2017;7(6):e014537.

9. Alvarez-Gallardo IC, Bidonde J, Busch A, et al. Therapeutic validity of exercise interventions in the management of fibromyalgia. *The Journal of sports medicine and physical fitness.* 2019;59(5):828-838.

10. Amini B, Beckmann NM, Beaman FD, et al. ACR Appropriateness Criteria((R)) Shoulder Pain-Traumatic. *Journal of the American College of Radiology : JACR.* 2018;15(5s):S171-s188.

11. Amiri Arimi S, Mohseni Bandpei MA, Javanshir K, Rezasoltani A, Biglarian A. The Effect of Different Exercise Programs on Size and Function of Deep Cervical Flexor Muscles in Patients With Chronic Nonspecific Neck Pain: A Systematic Review of Randomized Controlled Trials. *American journal of physical medicine & rehabilitation.* 2017;96(8):582-588.

12. Ananias J, Ubilla D, Irarrazaval S, Ortiz-Munoz L. Is pulsed ultrasound an alternative for osteoarthritis? *Medwave.* 2017;17(9):e7109.

13. Anwer S, Alghadir A, Zafar H, Brismee JM. Effects of orthopaedic manual therapy in knee osteoarthritis: a systematic review and meta-analysis. *Physiotherapy.* 2018;104(3):264-276.

14. Araujo FX, Scholl Schell M, Ribeiro DC. Effectiveness of Physiotherapy interventions plus Extrinsic Feedback for neck disorders: A systematic review with meta-analysis. *Musculoskeletal science & practice.* 2017;29:132-143.

15. Arshi A, Fabricant PD, Go DE, Williams RJ, McAllister DR, Jones KJ. Can Biologic Augmentation Improve Clinical Outcomes Following Microfracture for Symptomatic Cartilage Defects of the Knee? A Systematic Review. *Cartilage.* 2018;9(2):146-155.

16. Baez S, Hoch MC, Hoch JM. Evaluation of Cognitive Behavioral Interventions and Psychoeducation Implemented by Rehabilitation Specialists to Treat Fear-Avoidance Beliefs in Patients With Low Back Pain: A Systematic Review. *Archives of physical medicine and rehabilitation.* 2018;99(11):2287-2298.

17. Baird E. Interventions for treating persistent pain in survivors of torture. *Cochrane Database of Systematic Reviews.* 2019(2).

18. Baird E, Williams ACC, Hearn L, Amris K. Interventions for treating persistent pain in survivors of torture. *The Cochrane database of systematic reviews.* 2017;8:Cd012051.

19. Balasukumaran T, Olivier B, Ntsiea MV. The effectiveness of backward walking as a treatment for people with gait impairments: a systematic review and meta-analysis. *Clinical rehabilitation.* 2019;33(2):171-182.

20. Banerjee A, Hendrick P, Bhattacharjee P, Blake H. A systematic review of outcome measures utilised to assess self-management in clinical trials in patients with chronic pain. *Patient education and counseling.* 2018;101(5):767-778.

21. Barber-Westin S, Noyes FR. Blood Flow-Restricted Training for Lower Extremity Muscle Weakness due to Knee Pathology: A Systematic Review. *Sports health.* 2019;11(1):69-83.

22. Bartholdy C, Juhl C, Christensen R, Lund H, Zhang W, Henriksen M. The role of muscle strengthening in exercise therapy for knee osteoarthritis: A systematic review and meta-regression analysis of randomized trials. *Seminars in arthritis and rheumatism.* 2017;47(1):9-21.

23. Basson A, Olivier B, Ellis R, Coppieters M, Stewart A, Mudzi W. The Effectiveness of Neural Mobilization for Neuromusculoskeletal Conditions: A Systematic Review and Meta-analysis. *The Journal of orthopaedic and sports physical therapy.* 2017;47(9):593-615.

24. Bay S, Kuster L, McLean N, Byrnes M, Kuster MS. A systematic review of psychological interventions in total hip and knee arthroplasty. *BMC musculoskeletal disorders.* 2018;19(1):201.

25. Beck BR, Daly RM, Singh MA, Taaffe DR. Exercise and Sports Science Australia (ESSA) position statement on exercise prescription for the prevention and management of osteoporosis. *Journal of science and medicine in sport.* 2017;20(5):438-445.

26. Bernardy K, Klose P, Welsch P, Hauser W. Efficacy, acceptability and safety of cognitive behavioural therapies in fibromyalgia syndrome - A systematic review and meta-analysis of randomized controlled trials. *European journal of pain (London, England).* 2018;22(2):242-260.

27. Bernardy K, Klose P, Welsch P, Hauser W. Efficacy, acceptability and safety of Internet-delivered psychological therapies for fibromyalgia syndrome: A systematic review and meta-analysis of randomized controlled trials. *European journal of pain (London, England).* 2019;23(1):3-14.

28. Bhattarai P, Newton-John TRO, Phillips JL. Quality and Usability of Arthritic Pain Self-Management Apps for Older Adults: A Systematic Review. *Pain medicine (Malden, Mass).* 2018;19(3):471-484.

29. Bidonde J. Aerobic exercise training for adults with fibromyalgia. *Cochrane Database of Systematic Reviews.* 2017(6).

30. Bidonde J. Mixed exercise training for adults with fibromyalgia. *Cochrane Database of Systematic Reviews.* 2019(5).

31. Bidonde J, Busch AJ, Schachter CL, et al. Aerobic exercise training for adults with fibromyalgia. *The Cochrane database of systematic reviews.* 2017;6:Cd012700.

32. Bidonde J, Busch AJ, van der Spuy I, Tupper S, Kim SY, Boden C. Whole body vibration exercise training for fibromyalgia. *The Cochrane database of systematic reviews.* 2017;9:Cd011755.

33. Blomgren J, Strandell E, Jull G, Vikman I, Roijezon U. Effects of deep cervical flexor training on impaired physiological functions associated with chronic neck pain: a systematic review. *BMC musculoskeletal disorders.* 2018;19(1):415.

34. Bonvanie IJ, Kallesoe KH, Janssens KAM, Schroder A, Rosmalen JGM, Rask CU. Psychological Interventions for Children with Functional Somatic Symptoms: A Systematic Review and Meta-Analysis. *The Journal of pediatrics.* 2017;187:272-281.e217.

35. Bordeleau M, Carrondo Cottin S, Meier K, Prud'Homme M. Effects of Tonic Spinal Cord Stimulation on Sensory Perception in Chronic Pain Patients: A Systematic Review. *Neuromodulation : journal of the International Neuromodulation Society.* 2019;22(2):149-162.

36. Boyd A, Bleakley C, Hurley DA, et al. Herbal medicinal products or preparations for neuropathic pain. *The Cochrane database of systematic reviews.* 2019;4:Cd010528.

37. Briani RV, Ferreira AS, Pazzinatto MF, Pappas E, De Oliveira Silva D, Azevedo FM. What interventions can improve quality of life or psychosocial factors of individuals with knee osteoarthritis? A systematic review with meta-analysis of primary outcomes from randomised controlled trials. *British journal of sports medicine.* 2018;52(16):1031-1038.

38. Bricca A. Exercise does not 'wear down my knee': systematic reviews and meta-analyses. *British journal of sports medicine.* 2018;52(24):1591-1592.

39. Bright P, Hambly K. What Is the Proportion of Studies Reporting Patient and Practitioner Satisfaction with Software Support Tools Used in the Management of Knee Pain and Is This Related to Sample Size, Effect Size, and Journal Impact Factor? *Telemedicine journal and e-health : the official journal of the American Telemedicine Association.* 2018;24(8):562-576.

40. Brignardello-Petersen R, Guyatt GH, Buchbinder R, et al. Knee arthroscopy versus conservative management in patients with degenerative knee disease: a systematic review. *BMJ open.* 2017;7(5):e016114.

41. Brosseau L, Taki J, Desjardins B, et al. The Ottawa panel clinical practice guidelines for the management of knee osteoarthritis. Part one: introduction, and mind-body exercise programs. *Clinical rehabilitation.* 2017;31(5):582-595.

42. Brosseau L, Taki J, Desjardins B, et al. The Ottawa panel clinical practice guidelines for the management of knee osteoarthritis. Part two: strengthening exercise programs. *Clinical rehabilitation.* 2017;31(5):596-611.

43. Brosseau L, Taki J, Desjardins B, et al. The Ottawa panel clinical practice guidelines for the management of knee osteoarthritis. Part three: aerobic exercise programs. *Clinical rehabilitation.* 2017;31(5):612-624.

44. Brosseau L, Thevenot O, MacKiddie O, et al. The Ottawa Panel guidelines on programmes involving therapeutic exercise for the management of hand osteoarthritis. *Clinical rehabilitation.* 2018;32(11):1449-1471.

45. Byrnes K, Wu PJ, Whillier S. Is Pilates an effective rehabilitation tool? A systematic review. *Journal of bodywork and movement therapies.* 2018;22(1):192-202.

46. Cadalso RT, Jr., Daugherty J, Holmes C, Ram S, Enciso R. Efficacy of Electrical Stimulation of the Occipital Nerve in Intractable Primary Headache Disorders: A Systematic Review with Meta-Analyses. *Journal of oral & facial pain and headache.*32(1):40-52.

47. Cao Z, Mai X, Wang J, Feng E, Huang Y. Unicompartmental Knee Arthroplasty vs High Tibial Osteotomy for Knee Osteoarthritis: A Systematic Review and Meta-Analysis. *The Journal of arthroplasty.* 2018;33(3):952-959.

48. Ceballos-Laita L, Estebanez-de-Miguel E, Martin-Nieto G, Bueno-Gracia E, Fortun-Agud M, Jimenez-Del-Barrio S. Effects of non-pharmacological conservative treatment on pain, range of motion and physical function in patients with mild to moderate hip osteoarthritis. A systematic review. *Complementary therapies in medicine.* 2019;42:214-222.

49. Chang Chien GC, Mekhail N. Alternate Intraspinal Targets for Spinal Cord Stimulation: A Systematic Review. *Neuromodulation : journal of the International Neuromodulation Society.* 2017;20(7):629-641.

50. Charlesworth J, Fitzpatrick J, Perera NKP, Orchard J. Osteoarthritis- a systematic review of long-term safety implications for osteoarthritis of the knee. *BMC musculoskeletal disorders.* 2019;20(1):151.

51. Chen H, Li S, Ruan T, Liu L, Fang L. Is it necessary to perform prehabilitation exercise for patients undergoing total knee arthroplasty: meta-analysis of randomized controlled trials. *The Physician and sportsmedicine.* 2018;46(1):36-43.

52. Chen J, Li J, Li R, et al. Efficacy and Safety of Tanezumab on Osteoarthritis Knee and Hip Pains: A Meta-Analysis of Randomized Controlled Trials. *Pain medicine (Malden, Mass).* 2017;18(2):374-385.

53. Chen L, Lin CC, Huang TW, et al. Effect of acupuncture on aromatase inhibitor-induced arthralgia in patients with breast cancer: A meta-analysis of randomized controlled trials. *Breast (Edinburgh, Scotland).* 2017;33:132-138.

54. Chen N, Wang J, Mucelli A, Zhang X, Wang C. Electro-Acupuncture is Beneficial for Knee Osteoarthritis: The Evidence from Meta-Analysis of Randomized Controlled Trials. *The American journal of Chinese medicine.* 2017;45(5):965-985.

55. Chen X, Zou K, Abdullah N, et al. The placebo effect and its determinants in fibromyalgia: meta-analysis of randomised controlled trials. *Clinical rheumatology.* 2017;36(7):1623-1630.

56. Chesham RA, Shanmugam S. Does preoperative physiotherapy improve postoperative, patient-based outcomes in older adults who have undergone total knee arthroplasty? A systematic review. *Physiotherapy theory and practice.* 2017;33(1):9-30.

57. Cho JH, Lee JH, Song KS, et al. Treatment Outcomes for Patients with Failed Back Surgery. *Pain physician.* 2017;20(1):E29-e43.

58. Choi TY, Lee MS, Kim JI, Zaslawski C. Moxibustion for the treatment of osteoarthritis: An updated systematic review and meta-analysis. *Maturitas.* 2017;100:33-48.

59. Chou L, Ellis L, Papandony M, et al. Patients' perceived needs of osteoarthritis health information: A systematic scoping review. *PloS one.* 2018;13(4):e0195489.

60. Chou R, Deyo R, Friedly J, et al. Nonpharmacologic Therapies for Low Back Pain: A Systematic Review for an American College of Physicians Clinical Practice Guideline. *Annals of internal medicine.* 2017;166(7):493-505.

61. Chu IJH, Lim AYT, Ng CLW. Effects of meaningful weight loss beyond symptomatic relief in adults with knee osteoarthritis and obesity: a systematic review and meta-analysis. *Obesity reviews : an official journal of the International Association for the Study of Obesity.* 2018;19(11):1597-1607.

62. Cibrian K. Nondrug Interventions Reduce Pain and Opioid Use After Total Knee Arthroplasty. *The American journal of nursing.* 2017;117(11):62.

63. Cibulka MT, Bloom NJ, Enseki KR, Macdonald CW, Woehrle J, McDonough CM. Hip Pain and Mobility Deficits-Hip Osteoarthritis: Revision 2017. *The Journal of orthopaedic and sports physical therapy.* 2017;47(6):A1-a37.

64. Cimpianu CL, Strube W, Falkai P, Palm U, Hasan A. Vagus nerve stimulation in psychiatry: a systematic review of the available evidence. *Journal of neural transmission (Vienna, Austria : 1996).* 2017;124(1):145-158.

65. Clark J, Nijs J, Yeowell G, Goodwin PC. What Are the Predictors of Altered Central Pain Modulation in Chronic Musculoskeletal Pain Populations? A Systematic Review. *Pain physician.* 2017;20(6):487-500.

66. Coburn SL, Barton CJ, Filbay SR, Hart HF, Rathleff MS, Crossley KM. Quality of life in individuals with patellofemoral pain: A systematic review including meta-analysis. *Physical therapy in sport : official journal of the Association of Chartered Physiotherapists in Sports Medicine.* 2018;33:96-108.

67. Cohen EM, Morley-Fletcher A, Mehta DH, Lee YC. A systematic review of psychosocial therapies for children with rheumatic diseases. *Pediatric rheumatology online journal.* 2017;15(1):6.

68. Cole B, McGrath B, Salottolo K, Bar-Or D. LMWF-5A for the Treatment of Severe Osteoarthritis of the Knee: Integrated Analysis of Safety and Efficacy. *Orthopedics.* 2018;41(1):e77-e83.

69. Cooper TE, Wiffen PJ, Heathcote LC, et al. Antiepileptic drugs for chronic non-cancer pain in children and adolescents. *The Cochrane database of systematic reviews.* 2017;8:Cd012536.

70. Corbett M, South E, Harden M, et al. Brain and spinal stimulation therapies for phantom limb pain: a systematic review. *Health technology assessment (Winchester, England).* 2018;22(62):1-94.

71. Coulombe BJ, Games KE, Neil ER, Eberman LE. Core Stability Exercise Versus General Exercise for Chronic Low Back Pain. *Journal of athletic training.* 2017;52(1):71-72.

72. Coulter ID, Crawford C, Hurwitz EL, et al. Manipulation and mobilization for treating chronic low back pain: a systematic review and meta-analysis. *The spine journal : official journal of the North American Spine Society.* 2018;18(5):866-879.

73. Coulter ID, Crawford C, Vernon H, et al. Manipulation and Mobilization for Treating Chronic Nonspecific Neck Pain: A Systematic Review and Meta-Analysis for an Appropriateness Panel. *Pain physician.* 2019;22(2):E55-e70.

74. Cramer H, Klose P, Brinkhaus B, Michalsen A, Dobos G. Effects of yoga on chronic neck pain: a systematic review and meta-analysis. *Clinical rehabilitation.* 2017;31(11):1457-1465.

75. Cudejko T, van der Esch M, van der Leeden M, et al. Effect of Soft Braces on Pain and Physical Function in Patients With Knee Osteoarthritis: Systematic Review With Meta-Analyses. *Archives of physical medicine and rehabilitation.* 2018;99(1):153-163.

76. Cukiert A, Reis AMD, Almeida AS, Simoes RDS, Buzzini RF, Bernardo WM. Chronic pain treatment with spinal cord neurostimulation. *Revista da Associacao Medica Brasileira (1992).* 2018;64(4):299-306.

77. David M, Moraes AA, Costa MLD, Franco CIF. Transcranial direct current stimulation in the modulation of neuropathic pain: a systematic review. *Neurological research.* 2018;40(7):555-563.

78. Decary S, Ouellet P, Vendittoli PA, Roy JS, Desmeules F. Diagnostic validity of physical examination tests for common knee disorders: An overview of systematic reviews and meta-analysis. *Physical therapy in sport : official journal of the Association of Chartered Physiotherapists in Sports Medicine.* 2017;23:143-155.

79. Deer TR, Pope JE, Lamer TJ, et al. The Neuromodulation Appropriateness Consensus Committee on Best Practices for Dorsal Root Ganglion Stimulation. *Neuromodulation : journal of the International Neuromodulation Society.* 2019;22(1):1-35.

80. Del Grossi Moura M, Lopes LC, Biavatti MW, et al. Oral herbal medicines marketed in Brazil for the treatment of osteoarthritis: A systematic review and meta-analysis. *Phytotherapy research : PTR.* 2017;31(11):1676-1685.

81. Derry S, Wiffen PJ, Hauser W, et al. Oral nonsteroidal anti-inflammatory drugs for fibromyalgia in adults. *The Cochrane database of systematic reviews.* 2017;3:Cd012332.

82. Di Y, Han C, Zhao L, Ren Y. Is local platelet-rich plasma injection clinically superior to hyaluronic acid for treatment of knee osteoarthritis? A systematic review of randomized controlled trials. *Arthritis research & therapy.* 2018;20(1):128.

83. Doma K, Grant A, Morris J. The Effects of Balance Training on Balance Performance and Functional Outcome Measures Following Total Knee Arthroplasty: A Systematic Review and Meta-Analysis. *Sports medicine (Auckland, NZ).* 2018;48(10):2367-2385.

84. Dommerholt J, Hooks T, Chou LW, Finnegan M. A critical overview of the current myofascial pain literature - November 2018. *Journal of bodywork and movement therapies.* 2019;23(1):65-73.

85. Dong R, Wu Y, Xu S, et al. Is aquatic exercise more effective than land-based exercise for knee osteoarthritis? *Medicine.* 2018;97(52):e13823.

86. Dresler T, Caratozzolo S, Guldolf K, et al. Understanding the nature of psychiatric comorbidity in migraine: a systematic review focused on interactions and treatment implications. *The journal of headache and pain.* 2019;20(1):51.

87. Driban JB, Hootman JM, Sitler MR, Harris KP, Cattano NM. Is Participation in Certain Sports Associated With Knee Osteoarthritis? A Systematic Review. *Journal of athletic training.* 2017;52(6):497-506.

88. Eccleston C, Cooper TE, Fisher E, Anderson B, Wilkinson NM. Non-steroidal anti-inflammatory drugs (NSAIDs) for chronic non-cancer pain in children and adolescents. *The Cochrane database of systematic reviews.* 2017;8:Cd012537.

89. Egerton T, Diamond LE, Buchbinder R, Bennell KL, Slade SC. A systematic review and evidence synthesis of qualitative studies to identify primary care clinicians' barriers and enablers to the management of osteoarthritis. *Osteoarthritis and cartilage.* 2017;25(5):625-638.

90. Ehrenbrusthoff K, Ryan CG, Gruneberg C, Martin DJ. A systematic review and meta-analysis of the reliability and validity of sensorimotor measurement instruments in people with chronic low back pain. *Musculoskeletal science & practice.* 2018;35:73-83.

91. Eyles JP, Hunter DJ, Meneses SRF, et al. Instruments assessing attitudes toward or capability regarding self-management of osteoarthritis: a systematic review of measurement properties. *Osteoarthritis and cartilage.* 2017;25(8):1210-1222.

92. Fernandopulle S, Perry M, Manlapaz D, Jayakaran P. Effect of Land-Based Generic Physical Activity Interventions on Pain, Physical Function, and Physical Performance in Hip and Knee Osteoarthritis: A Systematic Review and Meta-Analysis. *American journal of physical medicine & rehabilitation.* 2017;96(11):773-792.

93. Fisher E, Law E, Dudeney J, Eccleston C, Palermo TM. Psychological therapies (remotely delivered) for the management of chronic and recurrent pain in children and adolescents. *The Cochrane database of systematic reviews.* 2019;4:Cd011118.

94. Fisher E, Law E, Dudeney J, Palermo TM, Stewart G, Eccleston C. Psychological therapies for the management of chronic and recurrent pain in children and adolescents. *The Cochrane database of systematic reviews.* 2018;9:Cd003968.

95. Florez-Garcia M, Garcia-Perez F, Curbelo R, et al. Efficacy and safety of home-based exercises versus individualized supervised outpatient physical therapy programs after total knee arthroplasty: a systematic review and meta-analysis. *Knee surgery, sports traumatology, arthroscopy : official journal of the ESSKA.* 2017;25(11):3340-3353.

96. Fraioli A, Mennuni G, Fontana M, et al. Efficacy of Spa Therapy, Mud-Pack Therapy, Balneotherapy, and Mud-Bath Therapy in the Management of Knee Osteoarthritis. A Systematic Review. *BioMed research international.* 2018;2018:1042576.

97. Franke H, Franke JD, Belz S, Fryer G. Osteopathic manipulative treatment for low back and pelvic girdle pain during and after pregnancy: A systematic review and meta-analysis. *Journal of bodywork and movement therapies.* 2017;21(4):752-762.

98. Fredin K, Loras H. Manual therapy, exercise therapy or combined treatment in the management of adult neck pain - A systematic review and meta-analysis. *Musculoskeletal science & practice.* 2017;31:62-71.

99. Furlan AD, Irvin E, Munhall C, et al. Rehabilitation service models for people with physical and/or mental disability living in low- and middle-income countries: A systematic review. *Journal of rehabilitation medicine.* 2018;50(6):487-498.

100. Galvin CR, Perriman DM, Newman PM, Lynch JT, Smith PN, Scarvell JM. Squatting, lunging and kneeling provided similar kinematic profiles in healthy knees-A systematic review and meta-analysis of the literature on deep knee flexion kinematics. *The Knee.* 2018;25(4):514-530.

101. Gavin JP, Immins T, Wainwright T. Stair negotiation as a rehabilitation intervention for enhancing recovery following total hip and knee replacement surgery. *International journal of orthopaedic and trauma nursing.* 2017;25:3-10.

102. Geenen R, Overman CL, Christensen R, et al. EULAR recommendations for the health professional's approach to pain management in inflammatory arthritis and osteoarthritis. *Annals of the rheumatic diseases.* 2018;77(6):797-807.

103. Ghazi C, Nyland J, Whaley R, Rogers T, Wera J, Henzman C. Social cognitive or learning theory use to improve self-efficacy in musculoskeletal rehabilitation: A systematic review and meta-analysis. *Physiotherapy theory and practice.* 2018;34(7):495-504.

104. Gibson W. Transcutaneous electrical nerve stimulation (TENS) for chronic pain - an overview of Cochrane Reviews. *Cochrane Database of Systematic Reviews.* 2019(4).

105. Gibson W, Wand BM, Meads C, Catley MJ, O'Connell NE. Transcutaneous electrical nerve stimulation (TENS) for chronic pain - an overview of Cochrane Reviews. *The Cochrane database of systematic reviews.* 2019;4:Cd011890.

106. Gohal C, Shanmugaraj A, Tate P, et al. Effectiveness of Valgus Offloading Knee Braces in the Treatment of Medial Compartment Knee Osteoarthritis: A Systematic Review. *Sports health.* 2018;10(6):500-514.

107. Goncalves JPB, Lucchetti G, Menezes PR, Vallada H. Complementary religious and spiritual interventions in physical health and quality of life: A systematic review of randomized controlled clinical trials. *PloS one.* 2017;12(10):e0186539.

108. Grandhi RK, Kaye AD, Abd-Elsayed A. Systematic Review of Radiofrequency Ablation and Pulsed Radiofrequency for Management of Cervicogenic Headaches. *Current pain and headache reports.* 2018;22(3):18.

109. Gregori D, Giacovelli G, Minto C, et al. Association of Pharmacological Treatments With Long-term Pain Control in Patients With Knee Osteoarthritis: A Systematic Review and Meta-analysis. *Jama.* 2018;320(24):2564-2579.

110. Griffin A, Leaver A, Moloney N. General Exercise Does Not Improve Long-Term Pain and Disability in Individuals With Whiplash-Associated Disorders: A Systematic Review. *The Journal of orthopaedic and sports physical therapy.* 2017;47(7):472-480.

111. Guan VX, Mobasheri A, Probst YC. A systematic review of osteoarthritis prevention and management with dietary phytochemicals from foods. *Maturitas.* 2019;122:35-43.

112. Guay J, Suresh S, Kopp S. The use of ultrasound guidance for perioperative neuraxial and peripheral nerve blocks in children. *The Cochrane database of systematic reviews.* 2019;2:Cd011436.

113. Gupta A, Huettner DP, Dukewich M. Comparative Effectiveness Review of Cooled Versus Pulsed Radiofrequency Ablation for the Treatment of Knee Osteoarthritis: A Systematic Review. *Pain physician.* 2017;20(3):155-171.

114. Hall A, Copsey B, Richmond H, et al. Effectiveness of Tai Chi for Chronic Musculoskeletal Pain Conditions: Updated Systematic Review and Meta-Analysis. *Physical therapy.* 2017;97(2):227-238.

115. Hall M, Castelein B, Wittoek R, Calders P, Van Ginckel A. Diet-induced weight loss alone or combined with exercise in overweight or obese people with knee osteoarthritis: A systematic review and meta-analysis. *Seminars in arthritis and rheumatism.* 2019;48(5):765-777.

116. Harris KP, Driban JB, Sitler MR, Cattano NM, Balasubramanian E, Hootman JM. Tibiofemoral Osteoarthritis After Surgical or Nonsurgical Treatment of Anterior Cruciate Ligament Rupture: A Systematic Review. *Journal of athletic training.* 2017;52(6):507-517.

117. Hart HF, Barton CJ, Khan KM, Riel H, Crossley KM. Is body mass index associated with patellofemoral pain and patellofemoral osteoarthritis? A systematic review and meta-regression and analysis. *British journal of sports medicine.* 2017;51(10):781-790.

118. Hart HF, Stefanik JJ, Wyndow N, Machotka Z, Crossley KM. The prevalence of radiographic and MRI-defined patellofemoral osteoarthritis and structural pathology: a systematic review and meta-analysis. *British journal of sports medicine.* 2017;51(16):1195-1208.

119. Hassan F, Murrell WD, Refalo A, Maffulli N. Alternatives to Biologics in Management of Knee Osteoarthritis: A Systematic Review. *Sports medicine and arthroscopy review.* 2018;26(2):79-85.

120. Hassan F, Trebinjac S, Murrell WD, Maffulli N. The effectiveness of prolotherapy in treating knee osteoarthritis in adults: a systematic review. *British medical bulletin.* 2017;122(1):91-108.

121. Helm Ii S, Simopoulos TT, Stojanovic M, Abdi S, El Terany MA. Effectiveness of Thermal Annular Procedures in Treating Discogenic Low Back Pain. *Pain physician.* 2017;20(6):447-470.

122. Heneghan NR, Smith R, Tyros I, Falla D, Rushton A. Thoracic dysfunction in whiplash associated disorders: A systematic review. *PloS one.* 2018;13(3):e0194235.

123. Herling SF. Interventions for preventing intensive care unit delirium in adults. *Cochrane Database of Systematic Reviews.* 2019(1).

124. Herling SF, Greve IE, Vasilevskis EE, et al. Interventions for preventing intensive care unit delirium in adults. *The Cochrane database of systematic reviews.* 2018;11:Cd009783.

125. Hidalgo B, Hall T, Bossert J, Dugeny A, Cagnie B, Pitance L. The efficacy of manual therapy and exercise for treating non-specific neck pain: A systematic review. *Journal of back and musculoskeletal rehabilitation.* 2017;30(6):1149-1169.

126. Holanda LJ, Silva PMM, Amorim TC, Lacerda MO, Simao CR, Morya E. Robotic assisted gait as a tool for rehabilitation of individuals with spinal cord injury: a systematic review. *Journal of neuroengineering and rehabilitation.* 2017;14(1):126.

127. Holden MA, Burke DL, Runhaar J, et al. Subgrouping and TargetEd Exercise pRogrammes for knee and hip OsteoArthritis (STEER OA): a systematic review update and individual participant data meta-analysis protocol. *BMJ open.* 2017;7(12):e018971.

128. Honda Y, Sakamoto J, Hamaue Y, et al. Effects of Physical-Agent Pain Relief Modalities for Fibromyalgia Patients: A Systematic Review and Meta-Analysis of Randomized Controlled Trials. *Pain research & management.* 2018;2018:2930632.

129. Honvo G, Reginster JY, Rannou F, et al. Safety of Intra-articular Hyaluronic Acid Injections in Osteoarthritis: Outcomes of a Systematic Review and Meta-Analysis. *Drugs & aging.* 2019;36(Suppl 1):101-127.

130. Hughes L, Paton B, Rosenblatt B, Gissane C, Patterson SD. Blood flow restriction training in clinical musculoskeletal rehabilitation: a systematic review and meta-analysis. *British journal of sports medicine.* 2017;51(13):1003-1011.

131. Hurley M. Exercise interventions and patient beliefs for people with hip, knee or hip and knee osteoarthritis: a mixed methods review. *Cochrane Database of Systematic Reviews.* 2018(4).

132. Hurley M, Dickson K, Hallett R, et al. Exercise interventions and patient beliefs for people with hip, knee or hip and knee osteoarthritis: a mixed methods review. *The Cochrane database of systematic reviews.* 2018;4:Cd010842.

133. Iijima H, Shimoura K, Aoyama T, Takahashi M. Biomechanical characteristics of stair ambulation in patients with knee OA: A systematic review with meta-analysis toward a better definition of clinical hallmarks. *Gait & posture.* 2018;62:191-201.

134. Imran TF, Malapero R, Qavi AH, et al. Efficacy of spinal cord stimulation as an adjunct therapy for chronic refractory angina pectoris. *International journal of cardiology.* 2017;227:535-542.

135. Inai T, Takabayashi T, Edama M, Kubo M. Evaluation of factors that affect hip moment impulse during gait: A systematic review. *Gait & posture.* 2018;61:488-492.

136. Jackson JL, Mancuso JM, Nickoloff S, Bernstein R, Kay C. Tricyclic and Tetracyclic Antidepressants for the Prevention of Frequent Episodic or Chronic Tension-Type Headache in Adults: A Systematic Review and Meta-Analysis. *Journal of general internal medicine.* 2017;32(12):1351-1358.

137. Jacobson JA, Roberts CC, Bencardino JT, et al. ACR Appropriateness Criteria((R)) Chronic Extremity Joint Pain-Suspected Inflammatory Arthritis. *Journal of the American College of Radiology : JACR.* 2017;14(5s):S81-s89.

138. Jansons PS, Haines TP, O'Brien L. Interventions to achieve ongoing exercise adherence for adults with chronic health conditions who have completed a supervised exercise program: systematic review and meta-analysis. *Clinical rehabilitation.* 2017;31(4):465-477.

139. Jeong HS, Lee SC, Jee H, Song JB, Chang HS, Lee SY. Proprioceptive Training and Outcomes of Patients With Knee Osteoarthritis: A Meta-Analysis of Randomized Controlled Trials. *Journal of athletic training.* 2019;54(4):418-428.

140. Jette AM. The Importance of Dose of a Rehabilitation Intervention. *Physical therapy.* 2017;97(11):1043.

141. Jia P, Tang L, Yu J, et al. Risk of bias and methodological issues in randomised controlled trials of acupuncture for knee osteoarthritis: a cross-sectional study. *BMJ open.* 2018;8(3):e019847.

142. Johnson MI. Transcutaneous electrical nerve stimulation (TENS) for fibromyalgia in adults. *Cochrane Database of Systematic Reviews.* 2017(10).

143. Johnson MI, Claydon LS, Herbison GP, Jones G, Paley CA. Transcutaneous electrical nerve stimulation (TENS) for fibromyalgia in adults. *The Cochrane database of systematic reviews.* 2017;10:Cd012172.

144. Johnston R, Cahalan R, O'Keeffe M, O'Sullivan K, Comyns T. The associations between training load and baseline characteristics on musculoskeletal injury and pain in endurance sport populations: A systematic review. *Journal of science and medicine in sport.* 2018;21(9):910-918.

145. Jonsson A, Rasmussen-Barr E. Intra- and inter-rater reliability of movement and palpation tests in patients with neck pain: A systematic review. *Physiotherapy theory and practice.* 2018;34(3):165-180.

146. Joustra ML, Minovic I, Janssens KAM, Bakker SJL, Rosmalen JGM. Vitamin and mineral status in chronic fatigue syndrome and fibromyalgia syndrome: A systematic review and meta-analysis. *PloS one.* 2017;12(4):e0176631.

147. Kahlaee AH, Ghamkhar L, Arab AM. The Association Between Neck Pain and Pulmonary Function: A Systematic Review. *American journal of physical medicine & rehabilitation.* 2017;96(3):203-210.

148. Kanavaki AM, Rushton A, Efstathiou N, et al. Barriers and facilitators of physical activity in knee and hip osteoarthritis: a systematic review of qualitative evidence. *BMJ open.* 2017;7(12):e017042.

149. Kanis JA, Cooper C, Rizzoli R, Reginster JY. Executive summary of European guidance for the diagnosis and management of osteoporosis in postmenopausal women. *Aging clinical and experimental research.* 2019;31(1):15-17.

150. Kapural L, Peterson E, Provenzano DA, Staats P. Clinical Evidence for Spinal Cord Stimulation for Failed Back Surgery Syndrome (FBSS): Systematic Review. *Spine.* 2017;42 Suppl 14:S61-s66.

151. Karpinski K, Muller-Rath R, Niemeyer P, Angele P, Petersen W. Subgroups of patients with osteoarthritis and medial meniscus tear or crystal arthropathy benefit from arthroscopic treatment. *Knee surgery, sports traumatology, arthroscopy : official journal of the ESSKA.* 2019;27(3):782-796.

152. Kelley GA, Kelley KS, Callahan LF. Community-deliverable exercise and anxiety in adults with arthritis and other rheumatic diseases: a systematic review with meta-analysis of randomised controlled trials. *BMJ open.* 2018;8(2):e019138.

153. Keshavarz R, Bashardoust Tajali S, Mir SM, Ashrafi H. The role of scapular kinematics in patients with different shoulder musculoskeletal disorders: A systematic review approach. *Journal of bodywork and movement therapies.* 2017;21(2):386-400.

154. Kim R, Wiest C, Clark K, Cook C, Horn M. Identifying risk factors for first-episode neck pain: A systematic review. *Musculoskeletal science & practice.* 2018;33:77-83.

155. Koenig I, Eichelberger P, Blasimann A, Hauswirth A, Baeyens JP, Radlinger L. Wavelet analyses of electromyographic signals derived from lower extremity muscles while walking or running: A systematic review. *PloS one.* 2018;13(11):e0206549.

156. Krsticevic M, Jeric M, Dosenovic S, Jelicic Kadic A, Puljak L. Proliferative injection therapy for osteoarthritis: a systematic review. *International orthopaedics.* 2017;41(4):671-679.

157. Kunutsor SK, Beswick AD, Peters TJ, et al. Health Care Needs and Support for Patients Undergoing Treatment for Prosthetic Joint Infection following Hip or Knee Arthroplasty: A Systematic Review. *PloS one.* 2017;12(1):e0169068.

158. Kwak SG, Lee DG, Chang MC. Effectiveness of pulsed radiofrequency treatment on cervical radicular pain: A meta-analysis. *Medicine.* 2018;97(31):e11761.

159. Laimi K, Makila A, Barlund E, et al. Effectiveness of myofascial release in treatment of chronic musculoskeletal pain: a systematic review. *Clinical rehabilitation.* 2018;32(4):440-450.

160. Lan L, Zhang X, Li X, Rong X, Peng Y. The efficacy of transcranial magnetic stimulation on migraine: a meta-analysis of randomized controlled trails. *The journal of headache and pain.* 2017;18(1):86.

161. Lane NE, Shidara K, Wise BL. Osteoarthritis year in review 2016: clinical. *Osteoarthritis and cartilage.* 2017;25(2):209-215.

162. Lee JK, Kim HJ, Park JO, Yang JH. Inferior outcome of revision of unicompartmental knee arthroplasty to total knee arthroplasty compared with primary total knee arthroplasty: systematic review and meta-analysis. *Knee surgery, sports traumatology, arthroscopy : official journal of the ESSKA.* 2018;26(11):3403-3418.

163. Lee OS, Ahn S, Ahn JH, Teo SH, Lee YS. Effectiveness of concurrent procedures during high tibial osteotomy for medial compartment osteoarthritis: a systematic review and meta-analysis. *Archives of orthopaedic and trauma surgery.* 2018;138(2):227-236.

164. Lee YH, Song GG. Association between low bone mineral density and fibromyalgia: a meta-analysis. *Clinical rheumatology.* 2017;36(11):2573-2579.

165. Lee YS, Howell SM, Won YY, et al. Kinematic alignment is a possible alternative to mechanical alignment in total knee arthroplasty. *Knee surgery, sports traumatology, arthroscopy : official journal of the ESSKA.* 2017;25(11):3467-3479.

166. Leite VF, Daud Amadera JE, Buehler AM. Viscosupplementation for Hip Osteoarthritis: A Systematic Review and Meta-Analysis of the Efficacy on Pain and Disability, and the Occurrence of Adverse Events. *Archives of physical medicine and rehabilitation.* 2018;99(3):574-583.e571.

167. Lemmon R, Hampton A. Nonpharmacologic treatment of chronic pain: What works? *J Fam Pract.* 2018;67(8):474;477;480;483.

168. Leopoldino AO, Machado GC, Ferreira PH, et al. Paracetamol versus placebo for knee and hip osteoarthritis. *The Cochrane database of systematic reviews.* 2019;2:Cd013273.

169. Li JQ, Guo W, Sun ZG, et al. Cupping therapy for treating knee osteoarthritis: The evidence from systematic review and meta-analysis. *Complementary therapies in clinical practice.* 2017;28:152-160.

170. Li X, Wang R, Xing X, et al. Acupuncture for Myofascial Pain Syndrome: A Network Meta-Analysis of 33 Randomized Controlled Trials. *Pain physician.* 2017;20(6):E883-e902.

171. Li Y, Li S, Jiang J, Yuan S. Effects of yoga on patients with chronic nonspecific neck pain: A PRISMA systematic review and meta-analysis. *Medicine.* 2019;98(8):e14649.

172. Li Y, Yin Y, Jia G, Chen H, Yu L, Wu D. Effects of kinesiotape on pain and disability in individuals with chronic low back pain: a systematic review and meta-analysis of randomized controlled trials. *Clinical rehabilitation.* 2019;33(4):596-606.

173. Liao CD, Tsauo JY, Huang SW, Chen HC, Chiu YS, Liou TH. Preoperative range of motion and applications of continuous passive motion predict outcomes after knee arthroplasty in patients with arthritis. *Knee surgery, sports traumatology, arthroscopy : official journal of the ESSKA.* 2019;27(4):1259-1269.

174. Liao J, Wang T, Dong W, et al. Acupoint injection for nonspecific chronic low back pain: A protocol of systematic review. *Medicine.* 2019;98(29):e16478.

175. Lin I, Wiles LK, Waller R, et al. Poor overall quality of clinical practice guidelines for musculoskeletal pain: a systematic review. *British journal of sports medicine.* 2018;52(5):337-343.

176. Lin YC, Wan L, Jamison RN. Using Integrative Medicine in Pain Management: An Evaluation of Current Evidence. *Anesth Analg.* 2017;125(6):2081-2093.

177. Liu X, Machado GC, Eyles JP, Ravi V, Hunter DJ. Dietary supplements for treating osteoarthritis: a systematic review and meta-analysis. *British journal of sports medicine.* 2018;52(3):167-175.

178. Liu YF, Kim Y, Yoo T, Han P, Inman JC. Burning mouth syndrome: a systematic review of treatments. *Oral diseases.* 2018;24(3):325-334.

179. Longo UG, Ciuffreda M, Rizzello G, Mannering N, Maffulli N, Denaro V. Surgical versus conservative management of Type III acromioclavicular dislocation: a systematic review. *British medical bulletin.* 2017;122(1):31-49.

180. Lue S, Koppikar S, Shaikh K, Mahendira D, Towheed TE. Systematic review of non-surgical therapies for osteoarthritis of the hand: an update. *Osteoarthritis and cartilage.* 2017;25(9):1379-1389.

181. Luo J, Yang L, Yang J, et al. Efficacy and safety of phosphodiesterase 4 inhibitors in patients with asthma: A systematic review and meta-analysis. *Respirology (Carlton, Vic).* 2018;23(5):467-477.

182. Luomajoki HA, Bonet Beltran MB, Careddu S, Bauer CM. Effectiveness of movement control exercise on patients with non-specific low back pain and movement control impairment: A systematic review and meta-analysis. *Musculoskeletal science & practice.* 2018;36:1-11.

183. Luque-Suarez A, Martinez-Calderon J, Falla D. Role of kinesiophobia on pain, disability and quality of life in people suffering from chronic musculoskeletal pain: a systematic review. *British journal of sports medicine.* 2019;53(9):554-559.

184. Luz Junior MAD, Almeida MO, Santos RS, Civile VT, Costa LOP. Effectiveness of Kinesio Taping in Patients With Chronic Nonspecific Low Back Pain: A Systematic Review With Meta-analysis. *Spine.* 2019;44(1):68-78.

185. MacPherson H, Vertosick EA, Foster NE, et al. The persistence of the effects of acupuncture after a course of treatment: a meta-analysis of patients with chronic pain. *Pain.* 2017;158(5):784-793.

186. Madaleno FO, Santos BA, Araujo VL, Oliveira VC, Resende RA. Prevalence of knee osteoarthritis in former athletes: a systematic review with meta-analysis. *Brazilian journal of physical therapy.* 2018;22(6):437-451.

187. Maffulli N, Longo UG, Locher J, Romeo G, Salvatore G, Denaro V. Outcome of ankle arthrodesis and ankle prosthesis: a review of the current status. *British medical bulletin.* 2017;124(1):91-112.

188. Magni NE, McNair PJ, Rice DA. The effects of resistance training on muscle strength, joint pain, and hand function in individuals with hand osteoarthritis: a systematic review and meta-analysis. *Arthritis research & therapy.* 2017;19(1):131.

189. Manchikanti L, Kaye AM, Knezevic NN, et al. Responsible, Safe, and Effective Prescription of Opioids for Chronic Non-Cancer Pain: American Society of Interventional Pain Physicians (ASIPP) Guidelines. *Pain physician.* 2017;20(2s):S3-s92.

190. Manheimer E. Acupuncture for hip osteoarthritis. *Cochrane Database of Systematic Reviews.* 2018(5).

191. Manheimer E, Cheng K, Wieland LS, et al. Acupuncture for hip osteoarthritis. *The Cochrane database of systematic reviews.* 2018;5:Cd013010.

192. Mansfield M, Thacker M, Spahr N, Smith T. Factors associated with physical activity participation in adults with chronic cervical spine pain: a systematic review. *Physiotherapy.* 2018;104(1):54-60.

193. Mariano TY, Urman RD, Hutchison CA, Jamison RN, Edwards RR. Cognitive Behavioral Therapy (CBT) for Subacute Low Back Pain: a Systematic Review. *Current pain and headache reports.* 2018;22(3):15.

194. Marin TJ, Van Eerd D, Irvin E, et al. Multidisciplinary biopsychosocial rehabilitation for subacute low back pain. *The Cochrane database of systematic reviews.* 2017;6:Cd002193.

195. Marley J, Tully MA, Porter-Armstrong A, et al. The effectiveness of interventions aimed at increasing physical activity in adults with persistent musculoskeletal pain: a systematic review and meta-analysis. *BMC musculoskeletal disorders.* 2017;18(1):482.

196. Matheve T, Brumagne S, Timmermans AAA. The Effectiveness of Technology-Supported Exercise Therapy for Low Back Pain: A Systematic Review. *American journal of physical medicine & rehabilitation.* 2017;96(5):347-356.

197. Matsumoto H, Hagino H, Hayashi K, et al. The effect of balneotherapy on pain relief, stiffness, and physical function in patients with osteoarthritis of the knee: a meta-analysis. *Clinical rheumatology.* 2017;36(8):1839-1847.

198. Maturen KE, Akin EA, Dassel M, et al. ACR Appropriateness Criteria((R)) Postmenopausal Subacute or Chronic Pelvic Pain. *Journal of the American College of Radiology : JACR.* 2018;15(11s):S365-s372.

199. May S, Runge N, Aina A. Centralization and directional preference: An updated systematic review with synthesis of previous evidence. *Musculoskeletal science & practice.* 2018;38:53-62.

200. McDowell CP, Cook DB, Herring MP. The Effects of Exercise Training on Anxiety in Fibromyalgia Patients: A Meta-analysis. *Medicine and science in sports and exercise.* 2017;49(9):1868-1876.

201. McEvoy C, Wiles L, Bernhardsson S, Grimmer K. Triage for Patients with Spinal Complaints: A Systematic Review of the Literature. *Physiotherapy research international : the journal for researchers and clinicians in physical therapy.* 2017;22(1).

202. Mendez-Rebolledo G, Gatica-Rojas V, Torres-Cueco R, Albornoz-Verdugo M, Guzman-Munoz E. Update on the effects of graded motor imagery and mirror therapy on complex regional pain syndrome type 1: A systematic review. *Journal of back and musculoskeletal rehabilitation.* 2017;30(3):441-449.

203. Meyer C, Denis CM, Berquin AD. Secondary prevention of chronic musculoskeletal pain: A systematic review of clinical trials. *Annals of physical and rehabilitation medicine.* 2018;61(5):323-338.

204. Minshull C, Gleeson N. Considerations of the Principles of Resistance Training in Exercise Studies for the Management of Knee Osteoarthritis: A Systematic Review. *Archives of physical medicine and rehabilitation.* 2017;98(9):1842-1851.

205. Miyamoto GC, Lin CC, Cabral CMN, van Dongen JM, van Tulder MW. Cost-effectiveness of exercise therapy in the treatment of non-specific neck pain and low back pain: a systematic review with meta-analysis. *British journal of sports medicine.* 2019;53(3):172-181.

206. Moens M, Goudman L, Brouns R, et al. Return to Work of Patients Treated With Spinal Cord Stimulation for Chronic Pain: A Systematic Review and Meta-Analysis. *Neuromodulation : journal of the International Neuromodulation Society.* 2019;22(3):253-261.

207. Moore M, Barker K. The validity and reliability of the four square step test in different adult populations: a systematic review. *Systematic reviews.* 2017;6(1):187.

208. Moraes LJ, Miranda MB, Loures LF, Mainieri AG, Marmora CHC. A systematic review of psychoneuroimmunology-based interventions. *Psychology, health & medicine.* 2018;23(6):635-652.

209. Morer C, Roques CF, Francon A, Forestier R, Maraver F. The role of mineral elements and other chemical compounds used in balneology: data from double-blind randomized clinical trials. *International journal of biometeorology.* 2017;61(12):2159-2173.

210. Moretti E, Tenorio A, Holanda L, Campos A, Lemos A. Efficacy of the whole-body vibration for pain, fatigue and quality of life in women with fibromyalgia: a systematic review. *Disability and rehabilitation.* 2018;40(9):988-996.

211. Moseng T, Dagfinrud H, Smedslund G, Osteras N. The importance of dose in land-based supervised exercise for people with hip osteoarthritis. A systematic review and meta-analysis. *Osteoarthritis and cartilage.* 2017;25(10):1563-1576.

212. Moura CC, Chaves ECL, Cardoso A, Nogueira DA, Correa HP, Chianca TCM. Cupping therapy and chronic back pain: systematic review and meta-analysis. *Revista latino-americana de enfermagem.* 2018;26:e3094.

213. Navani A, Manchikanti L, Albers SL, et al. Responsible, Safe, and Effective Use of Biologics in the Management of Low Back Pain: American Society of Interventional Pain Physicians (ASIPP) Guidelines. *Pain physician.* 2019;22(1s):S1-s74.

214. Nelson NL, Churilla JR. Massage Therapy for Pain and Function in Patients With Arthritis: A Systematic Review of Randomized Controlled Trials. *American journal of physical medicine & rehabilitation.* 2017;96(9):665-672.

215. Ng SK, Urquhart DM, Fitzgerald PB, Cicuttini FM, Hussain SM, Fitzgibbon BM. The Relationship Between Structural and Functional Brain Changes and Altered Emotion and Cognition in Chronic Low Back Pain Brain Changes: A Systematic Review of MRI and fMRI Studies. *The Clinical journal of pain.* 2018;34(3):237-261.

216. Nicolson PJA, Bennell KL, Dobson FL, Van Ginckel A, Holden MA, Hinman RS. Interventions to increase adherence to therapeutic exercise in older adults with low back pain and/or hip/knee osteoarthritis: a systematic review and meta-analysis. *British journal of sports medicine.* 2017;51(10):791-799.

217. O'Connell NE, Marston L, Spencer S, DeSouza LH, Wand BM. Non-invasive brain stimulation techniques for chronic pain. *The Cochrane database of systematic reviews.* 2018;4:Cd008208.

218. Onakpoya IJ, Spencer EA, Perera R, Heneghan CJ. Effectiveness of curcuminoids in the treatment of knee osteoarthritis: a systematic review and meta-analysis of randomized clinical trials. *International journal of rheumatic diseases.* 2017;20(4):420-433.

219. Orhurhu V, Urits I, Orman S, Viswanath O, Abd-Elsayed A. A Systematic Review of Radiofrequency Treatment of the Ankle for the Management of Chronic Foot and Ankle Pain. *Current pain and headache reports.* 2019;23(1):4.

220. Østerås N. Exercise for hand osteoarthritis. *Cochrane Database of Systematic Reviews.* 2017(1).

221. Osteras N, Kjeken I, Smedslund G, et al. Exercise for Hand Osteoarthritis: A Cochrane Systematic Review. *The Journal of rheumatology.* 2017;44(12):1850-1858.

222. Osteras N, Kjeken I, Smedslund G, et al. Exercise for hand osteoarthritis. *The Cochrane database of systematic reviews.* 2017;1:Cd010388.

223. Ouyang JH, Chang KH, Hsu WY, Cho YT, Liou TH, Lin YN. Non-elastic taping, but not elastic taping, provides benefits for patients with knee osteoarthritis: systemic review and meta-analysis. *Clinical rehabilitation.* 2018;32(1):3-17.

224. Overaas CK, Johansson MS, de Campos TF, et al. Prevalence and pattern of co-occurring musculoskeletal pain and its association with back-related disability among people with persistent low back pain: protocol for a systematic review and meta-analysis. *Systematic reviews.* 2017;6(1):258.

225. Pak J, Lee JH, Pak N, et al. Cartilage Regeneration in Humans with Adipose Tissue-Derived Stem Cells and Adipose Stromal Vascular Fraction Cells: Updated Status. *International journal of molecular sciences.* 2018;19(7).

226. Palmer JS. Surgical interventions for symptomatic mild to moderate knee osteoarthritis. *Cochrane Database of Systematic Reviews.* 2019(7).

227. Palmieri-Smith RM, Cameron KL, DiStefano LJ, et al. The Role of Athletic Trainers in Preventing and Managing Posttraumatic Osteoarthritis in Physically Active Populations: a Consensus Statement of the Athletic Trainers' Osteoarthritis Consortium. *Journal of athletic training.* 2017;52(6):610-623.

228. Panken G, Verhagen AP, Terwee CB, Heymans MW. Clinical Prediction Models for Patients With Nontraumatic Knee Pain in Primary Care: A Systematic Review and Internal Validation Study. *The Journal of orthopaedic and sports physical therapy.* 2017;47(8):518-529.

229. Papalia R, Diaz LA, Torre G, et al. Intrarticular injections of hyaluronic acid for trapezio-metacarpal osteoarthritis: a systematic review. *Journal of biological regulators and homeostatic agents.* 2017;31(4 Suppl 2):45-53.

230. Papandony MC, Chou L, Seneviwickrama M, et al. Patients' perceived health service needs for osteoarthritis (OA) care: a scoping systematic review. *Osteoarthritis and cartilage.* 2017;25(7):1010-1025.

231. Parreira P, Heymans MW, van Tulder MW, et al. Back Schools for chronic non-specific low back pain. *The Cochrane database of systematic reviews.* 2017;8:Cd011674.

232. Pas H, Moen MH, Haisma HJ, Winters M. No evidence for the use of stem cell therapy for tendon disorders: a systematic review. *British journal of sports medicine.* 2017;51(13):996-1002.

233. Pas R, Ickmans K, Van Oosterwijck S, et al. Hyperexcitability of the Central Nervous System in Children with Chronic Pain: A Systematic Review. *Pain medicine (Malden, Mass).* 2018;19(12):2504-2514.

234. Perry R, Leach V, Davies P, Penfold C, Ness A, Churchill R. An overview of systematic reviews of complementary and alternative therapies for fibromyalgia using both AMSTAR and ROBIS as quality assessment tools. *Systematic reviews.* 2017;6(1):97.

235. Phang JK, Kwan YH, Goh H, et al. Complementary and alternative medicine for rheumatic diseases: A systematic review of randomized controlled trials. *Complementary therapies in medicine.* 2018;37:143-157.

236. Poole JL, Siegel P. Effectiveness of Occupational Therapy Interventions for Adults With Fibromyalgia: A Systematic Review. *The American journal of occupational therapy : official publication of the American Occupational Therapy Association.* 2017;71(1):7101180040p7101180041-7101180040p7101180010.

237. Pozzobon D, Ferreira PH, Blyth FM, Machado GC, Ferreira ML. Can obesity and physical activity predict outcomes of elective knee or hip surgery due to osteoarthritis? A meta-analysis of cohort studies. *BMJ open.* 2018;8(2):e017689.

238. Probyn K, Bowers H, Mistry D, et al. Non-pharmacological self-management for people living with migraine or tension-type headache: a systematic review including analysis of intervention components. *BMJ open.* 2017;7(8):e016670.

239. Puljak L, Marin A, Vrdoljak D, Markotic F, Utrobicic A, Tugwell P. Celecoxib for osteoarthritis. *The Cochrane database of systematic reviews.* 2017;5:Cd009865.

240. Qaseem A, Wilt TJ, McLean RM, Forciea MA. Noninvasive Treatments for Acute, Subacute, and Chronic Low Back Pain: A Clinical Practice Guideline From the American College of Physicians. *Annals of internal medicine.* 2017;166(7):514-530.

241. Rabago D, Nourani B. Prolotherapy for Osteoarthritis and Tendinopathy: a Descriptive Review. *Current rheumatology reports.* 2017;19(6):34.

242. Ran J, Yang X, Ren Z, Wang J, Dong H. Comparison of intra-articular hyaluronic acid and methylprednisolone for pain management in knee osteoarthritis: A meta-analysis of randomized controlled trials. *International journal of surgery (London, England).* 2018;53:103-110.

243. Rausch Osthoff AK, Niedermann K, Braun J, et al. 2018 EULAR recommendations for physical activity in people with inflammatory arthritis and osteoarthritis. *Annals of the rheumatic diseases.* 2018;77(9):1251-1260.

244. Reginster JL, Arden NK, Haugen IK, et al. Guidelines for the conduct of pharmacological clinical trials in hand osteoarthritis: Consensus of a Working Group of the European Society on Clinical and Economic Aspects of Osteoporosis, Osteoarthritis and Musculoskeletal Diseases (ESCEO). *Seminars in arthritis and rheumatism.* 2018;48(1):1-8.

245. Reis F, Guimaraes F, Nogueira LC, Meziat-Filho N, Sanchez TA, Wideman T. Association between pain drawing and psychological factors in musculoskeletal chronic pain: A systematic review. *Physiotherapy theory and practice.* 2019;35(6):533-542.

246. Resende L, Merriwether E, Rampazo EP, et al. Meta-analysis of transcutaneous electrical nerve stimulation for relief of spinal pain. *European journal of pain (London, England).* 2018;22(4):663-678.

247. Richards R, van den Noort JC, Dekker J, Harlaar J. Gait Retraining With Real-Time Biofeedback to Reduce Knee Adduction Moment: Systematic Review of Effects and Methods Used. *Archives of physical medicine and rehabilitation.* 2017;98(1):137-150.

248. Rihn JA, Radcliff K, Norvell DC, et al. Comparative Effectiveness of Treatments for Chronic Low Back Pain: A Multiple Treatment Comparison Analysis. *Clinical spine surgery.* 2017;30(5):204-225.

249. Rodriguez-Merchan EC. Topical therapies for knee osteoarthritis. *Postgraduate medicine.* 2018;130(7):607-612.

250. Rubin DA, Roberts CC, Bencardino JT, et al. ACR Appropriateness Criteria((R)) Chronic Wrist Pain. *Journal of the American College of Radiology : JACR.* 2018;15(5s):S39-s55.

251. Rubinstein SM, de Zoete A, van Middelkoop M, Assendelft WJJ, de Boer MR, van Tulder MW. Benefits and harms of spinal manipulative therapy for the treatment of chronic low back pain: systematic review and meta-analysis of randomised controlled trials. *BMJ (Clinical research ed).* 2019;364:l689.

252. Ruiz Iban MA, Tejedor A, Gil Garay E, et al. GEDOS-SECOT consensus on the care process of patients with knee osteoarthritis and arthoplasty. *Revista espanola de cirugia ortopedica y traumatologia.* 2017;61(5):296-312.

253. Salamh P, Cook C, Reiman MP, Sheets C. Treatment effectiveness and fidelity of manual therapy to the knee: A systematic review and meta-analysis. *Musculoskeletal care.* 2017;15(3):238-248.

254. Salazar AP, Stein C, Marchese RR, Plentz RD, Pagnussat AS. Electric Stimulation for Pain Relief in Patients with Fibromyalgia: A Systematic Review and Meta-analysis of Randomized Controlled Trials. *Pain physician.* 2017;20(2):15-25.

255. Saltychev M, Laimi K. Effectiveness of repetitive transcranial magnetic stimulation in patients with fibromyalgia: a meta-analysis. *International journal of rehabilitation research Internationale Zeitschrift fur Rehabilitationsforschung Revue internationale de recherches de readaptation.* 2017;40(1):11-18.

256. Sansosti LE, Van JC, Meyr AJ. Effect of Obesity on Total Ankle Arthroplasty: A Systematic Review of Postoperative Complications Requiring Surgical Revision. *The Journal of foot and ankle surgery : official publication of the American College of Foot and Ankle Surgeons.* 2018;57(2):353-356.

257. Sanz-Banos Y, Pastor-Mira MA, Lledo A, Lopez-Roig S, Penacoba C, Sanchez-Meca J. Do women with fibromyalgia adhere to walking for exercise programs to improve their health? Systematic review and meta-analysis. *Disability and rehabilitation.* 2018;40(21):2475-2487.

258. Sattler LN, Hing WA, Vertullo CJ. What is the evidence to support early supervised exercise therapy after primary total knee replacement? A systematic review and meta-analysis. *BMC musculoskeletal disorders.* 2019;20(1):42.

259. Scerbo T, Colasurdo J, Dunn S, Unger J, Nijs J, Cook C. Measurement Properties of the Central Sensitization Inventory: A Systematic Review. *Pain practice : the official journal of World Institute of Pain.* 2018;18(4):544-554.

260. Schafer AGM, Zalpour C, von Piekartz H, Hall TM, Paelke V. The Efficacy of Electronic Health-Supported Home Exercise Interventions for Patients With Osteoarthritis of the Knee: Systematic Review. *Journal of medical Internet research.* 2018;20(4):e152.

261. Senftleber NK, Nielsen SM, Andersen JR, et al. Marine Oil Supplements for Arthritis Pain: A Systematic Review and Meta-Analysis of Randomized Trials. *Nutrients.* 2017;9(1).

262. Seo SY, Lee KB, Shin JS, et al. Effectiveness of Acupuncture and Electroacupuncture for Chronic Neck Pain: A Systematic Review and Meta-Analysis. *The American journal of Chinese medicine.* 2017;45(8):1573-1595.

263. Sepulveda F, Sanchez L, Amy E, Micheo W. Anterior Cruciate Ligament Injury: Return to Play, Function and Long-Term Considerations. *Current sports medicine reports.* 2017;16(3):172-178.

264. Shaw KE, Charlton JM, Perry CKL, et al. The effects of shoe-worn insoles on gait biomechanics in people with knee osteoarthritis: a systematic review and meta-analysis. *British journal of sports medicine.* 2018;52(4):238-253.

265. Shekelle PG, Cook IA, Miake-Lye IM, Booth MS, Beroes JM, Mak S. Benefits and Harms of Cranial Electrical Stimulation for Chronic Painful Conditions, Depression, Anxiety, and Insomnia: A Systematic Review. *Annals of internal medicine.* 2018;168(6):414-421.

266. Shen L, Yuan T, Chen S, Xie X, Zhang C. The temporal effect of platelet-rich plasma on pain and physical function in the treatment of knee osteoarthritis: systematic review and meta-analysis of randomized controlled trials. *Journal of orthopaedic surgery and research.* 2017;12(1):16.

267. Shin YS, Lee HN, Sim HB, Kim HJ, Lee DH. Polyurethane meniscal scaffolds lead to better clinical outcomes but worse articular cartilage status and greater absolute meniscal extrusion. *Knee surgery, sports traumatology, arthroscopy : official journal of the ESSKA.* 2018;26(8):2227-2238.

268. Shiri R, Falah-Hassani K. Does leisure time physical activity protect against low back pain? Systematic review and meta-analysis of 36 prospective cohort studies. *British journal of sports medicine.* 2017;51(19):1410-1418.

269. Shukla H, Nair SR, Thakker D. Role of telerehabilitation in patients following total knee arthroplasty: Evidence from a systematic literature review and meta-analysis. *Journal of telemedicine and telecare.* 2017;23(2):339-346.

270. Sielski R, Rief W, Glombiewski JA. Efficacy of Biofeedback in Chronic back Pain: a Meta-Analysis. *International journal of behavioral medicine.* 2017;24(1):25-41.

271. Sitthipornvorakul E, Klinsophon T, Sihawong R, Janwantanakul P. The effects of walking intervention in patients with chronic low back pain: A meta-analysis of randomized controlled trials. *Musculoskeletal science & practice.* 2018;34:38-46.

272. Skarstein S, Lagerlov P, Helseth S, Leegaard M. How do parents influence their adolescents' use of over-the-counter analgesics: A review of the current literature. *Journal of clinical nursing.* 2019;28(9-10):1451-1464.

273. Slater LV, Hart JM, Kelly AR, Kuenze CM. Progressive Changes in Walking Kinematics and Kinetics After Anterior Cruciate Ligament Injury and Reconstruction: A Review and Meta-Analysis. *Journal of athletic training.* 2017;52(9):847-860.

274. Smeraglia F, Mariconda M, Balato G, Di Donato SL, Criscuolo G, Maffulli N. Dubious space for Artelon joint resurfacing for basal thumb (trapeziometacarpal joint) osteoarthritis. A systematic review. *British medical bulletin.* 2018;126(1):79-84.

275. Sosa-Reina MD, Nunez-Nagy S, Gallego-Izquierdo T, Pecos-Martin D, Monserrat J, Alvarez-Mon M. Effectiveness of Therapeutic Exercise in Fibromyalgia Syndrome: A Systematic Review and Meta-Analysis of Randomized Clinical Trials. *BioMed research international.* 2017;2017:2356346.

276. Stenneberg MS, Rood M, de Bie R, Schmitt MA, Cattrysse E, Scholten-Peeters GG. To What Degree Does Active Cervical Range of Motion Differ Between Patients With Neck Pain, Patients With Whiplash, and Those Without Neck Pain? A Systematic Review and Meta-Analysis. *Archives of physical medicine and rehabilitation.* 2017;98(7):1407-1434.

277. Stitik TP, Issac SM, Modi S, Nasir S, Kulinets I. Effectiveness of 3 Weekly Injections Compared With 5 Weekly Injections of Intra-Articular Sodium Hyaluronate on Pain Relief of Knee Osteoarthritis or 3 Weekly Injections of Other Hyaluronan Products: A Systematic Review and Meta-Analysis. *Archives of physical medicine and rehabilitation.* 2017;98(5):1042-1050.

278. Stockings E, Campbell G, Hall WD, et al. Cannabis and cannabinoids for the treatment of people with chronic noncancer pain conditions: a systematic review and meta-analysis of controlled and observational studies. *Pain.* 2018;159(10):1932-1954.

279. Strom J, Bjerrum MB, Nielsen CV, et al. Anxiety and depression in spine surgery-a systematic integrative review. *The spine journal : official journal of the North American Spine Society.* 2018;18(7):1272-1285.

280. Takahashi T, Ansari J, Pandit HG. Kinematically Aligned Total Knee Arthroplasty or Mechanically Aligned Total Knee Arthroplasty. *The journal of knee surgery.* 2018;31(10):999-1006.

281. Tamin TZ, Murdana N, Pitoyo Y, Safitri ED. Exercise Intervention for Chronic Pain Management, Muscle Strengthening, and Functional Score in Obese Patients with Chronic Musculoskeletal Pain: A Systematic Review and Meta-analysis. *Acta medica Indonesiana.* 2018;50(4):299-308.

282. Tao H, Wang T, Dong X, Guo Q, Xu H, Wan Q. Effectiveness of transcutaneous electrical nerve stimulation for the treatment of migraine: a meta-analysis of randomized controlled trials. *The journal of headache and pain.* 2018;19(1):42.

283. Telfer S, Lange MJ, Sudduth ASM. Factors influencing knee adduction moment measurement: A systematic review and meta-regression analysis. *Gait & posture.* 2017;58:333-339.

284. Thabrew H. E-Health interventions for anxiety and depression in children and adolescents with long-term physical conditions. *Cochrane Database of Systematic Reviews.* 2018(8).

285. Thabrew H, Stasiak K, Hetrick SE, Wong S, Huss JH, Merry SN. E-Health interventions for anxiety and depression in children and adolescents with long-term physical conditions. *The Cochrane database of systematic reviews.* 2018;8:Cd012489.

286. Thieme K, Mathys M, Turk DC. Evidenced-Based Guidelines on the Treatment of Fibromyalgia Patients: Are They Consistent and If Not, Why Not? Have Effective Psychological Treatments Been Overlooked? *The journal of pain : official journal of the American Pain Society.* 2017;18(7):747-756.

287. Tian K, Cheng H, Zhang J, Chen K. Intra-articular injection of methylprednisolone for reducing pain in knee osteoarthritis: A systematic review and meta-analysis. *Medicine.* 2018;97(15):e0240.

288. Tick H, Nielsen A, Pelletier KR, et al. Evidence-Based Nonpharmacologic Strategies for Comprehensive Pain Care: The Consortium Pain Task Force White Paper. *Explore (NY).* 2018;14(3):177-211.

289. Timmins KA, Leech RD, Batt ME, Edwards KL. Running and Knee Osteoarthritis: A Systematic Review and Meta-analysis. *The American journal of sports medicine.* 2017;45(6):1447-1457.

290. Toivonen KI, Zernicke K, Carlson LE. Web-Based Mindfulness Interventions for People With Physical Health Conditions: Systematic Review. *Journal of medical Internet research.* 2017;19(8):e303.

291. Toupin April K, Bisaillon J, Welch V, et al. Tramadol for osteoarthritis. *The Cochrane database of systematic reviews.* 2019;5:Cd005522.

292. Tran G, Cowling P, Smith T, et al. What Imaging-Detected Pathologies Are Associated With Shoulder Symptoms and Their Persistence? A Systematic Literature Review. *Arthritis care & research.* 2018;70(8):1169-1184.

293. Umehara T, Tanaka R. Effective exercise intervention period for improving body function or activity in patients with knee osteoarthritis undergoing total knee arthroplasty: a systematic review and meta-analysis. *Brazilian journal of physical therapy.* 2018;22(4):265-275.

294. Upala S, Yong WC, Sanguankeo A. Bone mineral density is decreased in fibromyalgia syndrome: a systematic review and meta-analysis. *Rheumatology international.* 2017;37(4):617-622.

295. Usichenko T, Hacker H, Lotze M. Transcutaneous auricular vagal nerve stimulation (taVNS) might be a mechanism behind the analgesic effects of auricular acupuncture. *Brain stimulation.* 2017;10(6):1042-1044.

296. van der Straaten R, De Baets L, Jonkers I, Timmermans A. Mobile assessment of the lower limb kinematics in healthy persons and in persons with degenerative knee disorders: A systematic review. *Gait & posture.* 2018;59:229-241.

297. van Erp RMA, Huijnen IPJ, Jakobs MLG, Kleijnen J, Smeets R. Effectiveness of Primary Care Interventions Using a Biopsychosocial Approach in Chronic Low Back Pain: A Systematic Review. *Pain practice : the official journal of World Institute of Pain.* 2019;19(2):224-241.

298. Vigdorchik JM, Nepple JJ, Eftekhary N, Leunig M, Clohisy JC. What Is the Association of Elite Sporting Activities With the Development of Hip Osteoarthritis? *The American journal of sports medicine.* 2017;45(4):961-964.

299. Wajon A, Vinycomb T, Carr E, Edmunds I, Ada L. WITHDRAWN: Surgery for thumb (trapeziometacarpal joint) osteoarthritis. *The Cochrane database of systematic reviews.* 2017;4:Cd004631.

300. Waldstein W, Kolbitsch P, Koller U, Boettner F, Windhager R. Sport and physical activity following unicompartmental knee arthroplasty: a systematic review. *Knee surgery, sports traumatology, arthroscopy : official journal of the ESSKA.* 2017;25(3):717-728.

301. Wang H, Zhang C, Gao C, et al. Effects of short-wave therapy in patients with knee osteoarthritis: a systematic review and meta-analysis. *Clinical rehabilitation.* 2017;31(5):660-671.

302. Wang Y, Lu S, Wang R, et al. Integrative effect of yoga practice in patients with knee arthritis: A PRISMA-compliant meta-analysis. *Medicine.* 2018;97(31):e11742.

303. Welsch P. Mirtazapine for fibromyalgia in adults. *Cochrane Database of Systematic Reviews.* 2018(8).

304. Westad K, Tjoestolvsen F, Hebron C. The effectiveness of Mulligan's mobilisation with movement (MWM) on peripheral joints in musculoskeletal (MSK) conditions: A systematic review. *Musculoskeletal science & practice.* 2019;39:157-163.

305. Wewege MA, Booth J, Parmenter BJ. Aerobic vs. resistance exercise for chronic non-specific low back pain: A systematic review and meta-analysis. *Journal of back and musculoskeletal rehabilitation.* 2018;31(5):889-899.

306. Whittaker JL, Booysen N, de la Motte S, et al. Predicting sport and occupational lower extremity injury risk through movement quality screening: a systematic review. *British journal of sports medicine.* 2017;51(7):580-585.

307. Wieckiewicz M, Grychowska N, Zietek M, Wieckiewicz G, Smardz J. Evidence to Use Botulinum Toxin Injections in Tension-Type Headache Management: A Systematic Review. *Toxins.* 2017;9(11).

308. Wieland LS. Yoga treatment for chronic non-specific low back pain. *Cochrane Database of Systematic Reviews.* 2017(1).

309. Wieland LS, Skoetz N, Pilkington K, Vempati R, D'Adamo CR, Berman BM. Yoga treatment for chronic non-specific low back pain. *The Cochrane database of systematic reviews.* 2017;1:Cd010671.

310. Wijnen A, Bouma SE, Seeber GH, et al. The therapeutic validity and effectiveness of physiotherapeutic exercise following total hip arthroplasty for osteoarthritis: A systematic review. *PloS one.* 2018;13(3):e0194517.

311. Williams A, Kamper SJ, Wiggers JH, et al. Musculoskeletal conditions may increase the risk of chronic disease: a systematic review and meta-analysis of cohort studies. *BMC medicine.* 2018;16(1):167.

312. Winnard A, Nasser M, Debuse D, et al. Systematic review of countermeasures to minimise physiological changes and risk of injury to the lumbopelvic area following long-term microgravity. *Musculoskeletal science & practice.* 2017;27 Suppl 1:S5-s14.

313. Withers TM, Lister S, Sackley C, Clark A, Smith TO. Is there a difference in physical activity levels in patients before and up to one year after unilateral total hip replacement? A systematic review and meta-analysis. *Clinical rehabilitation.* 2017;31(5):639-650.

314. Wong JJ, Cote P, Sutton DA, et al. Clinical practice guidelines for the noninvasive management of low back pain: A systematic review by the Ontario Protocol for Traffic Injury Management (OPTIMa) Collaboration. *European journal of pain (London, England).* 2017;21(2):201-216.

315. Wood L, Hendrick PA. A systematic review and meta-analysis of pain neuroscience education for chronic low back pain: Short-and long-term outcomes of pain and disability. *European journal of pain (London, England).* 2019;23(2):234-249.

316. Woods B, Manca A, Weatherly H, et al. Cost-effectiveness of adjunct non-pharmacological interventions for osteoarthritis of the knee. *PloS one.* 2017;12(3):e0172749.

317. Wortman MSH, Lokkerbol J, van der Wouden JC, Visser B, van der Horst HE, Olde Hartman TC. Cost-effectiveness of interventions for medically unexplained symptoms: A systematic review. *PloS one.* 2018;13(10):e0205278.

318. Wouters RM, Tsehaie J, Hovius SER, Dilek B, Selles RW. Postoperative Rehabilitation Following Thumb Base Surgery: A Systematic Review of the Literature. *Archives of physical medicine and rehabilitation.* 2018;99(6):1177-1212.e1172.

319. Wshah A, Guilcher SJ, Goldstein R, Brooks D. Prevalence of osteoarthritis in individuals with COPD: a systematic review. *International journal of chronic obstructive pulmonary disease.* 2018;13:1207-1216.

320. Wu LC, Weng PW, Chen CH, Huang YY, Tsuang YH, Chiang CJ. Literature Review and Meta-Analysis of Transcutaneous Electrical Nerve Stimulation in Treating Chronic Back Pain. *Regional anesthesia and pain medicine.* 2018;43(4):425-433.

321. Wylde V, Dennis J, Beswick AD, et al. Systematic review of management of chronic pain after surgery. *The British journal of surgery.* 2017;104(10):1293-1306.

322. Wyszynska J, Bal-Bochenska M. Efficacy of High-Intensity Laser Therapy in Treating Knee Osteoarthritis: A First Systematic Review. *Photomedicine and laser surgery.* 2018;36(7):343-353.

323. Xu Q, Chen B, Wang Y, et al. The Effectiveness of Manual Therapy for Relieving Pain, Stiffness, and Dysfunction in Knee Osteoarthritis: A Systematic Review and Meta-Analysis. *Pain physician.* 2017;20(4):229-243.

324. Xu T, Zhou S, Zhang Y, et al. Acupuncture for chronic uncomplicated musculoskeletal pain associated with the spine: A systematic review protocol. *Medicine.* 2019;98(2):e14055.

325. Yang M, Jiang L, Wang Q, Chen H, Xu G. Traditional Chinese medicine for knee osteoarthritis: An overview of systematic review. *PloS one.* 2017;12(12):e0189884.

326. Yeganeh M, Baradaran HR, Qorbani M, Moradi Y, Dastgiri S. The effectiveness of acupuncture, acupressure and chiropractic interventions on treatment of chronic nonspecific low back pain in Iran: A systematic review and meta-analysis. *Complementary therapies in clinical practice.* 2017;27:11-18.

327. Yoo JI, Cha YH, Kim KJ, Kim HY, Choy WS, Hwang SC. Gait analysis after total hip arthroplasty using direct anterior approach versus anterolateral approach: a systematic review and meta-analysis. *BMC musculoskeletal disorders.* 2019;20(1):63.

328. Young JL, Rhon DI, Cleland JA, Snodgrass SJ. The Influence of Exercise Dosing on Outcomes in Patients With Knee Disorders: A Systematic Review. *The Journal of orthopaedic and sports physical therapy.* 2018;48(3):146-161.

329. Zech N, Hansen E, Bernardy K, Hauser W. Efficacy, acceptability and safety of guided imagery/hypnosis in fibromyalgia - A systematic review and meta-analysis of randomized controlled trials. *European journal of pain (London, England).* 2017;21(2):217-227.

330. Zhang J, Wang Q, Zhang C. Ineffectiveness of lateral-wedge insoles on the improvement of pain and function for medial knee osteoarthritis: a meta-analysis of controlled randomized trials. *Archives of orthopaedic and trauma surgery.* 2018;138(10):1453-1462.

331. Zhang L, Fu T, Zhang Q, et al. Effects of psychological interventions for patients with osteoarthritis: a systematic review and meta-analysis. *Psychology, health & medicine.* 2018;23(1):1-17.

332. Zhang Q, Yue J, Golianu B, Sun Z, Lu Y. Updated systematic review and meta-analysis of acupuncture for chronic knee pain. *Acupuncture in medicine : journal of the British Medical Acupuncture Society.* 2017;35(6):392-403.

333. Zhang X, Zhang Z, Wen J, Lu J, Sun Y, Sang D. The effectiveness of therapeutic strategies for patients with radiculopathy: A network meta-analysis. *Molecular pain.* 2018;14:1744806918768972.

334. Zhang Y, Huang L, Su Y, Zhan Z, Li Y, Lai X. The Effects of Traditional Chinese Exercise in Treating Knee Osteoarthritis: A Systematic Review and Meta-Analysis. *PloS one.* 2017;12(1):e0170237.

335. Zhou XY, Zhang XX, Yu GY, et al. Effects of Low-Intensity Pulsed Ultrasound on Knee Osteoarthritis: A Meta-Analysis of Randomized Clinical Trials. *BioMed research international.* 2018;2018:7469197.

336. Zhu CE, Yu B, Zhang W, Chen WH, Qi Q, Miao Y. Effiectiveness and safety of transcranial direct current stimulation in fibromyalgia: A systematic review and meta-analysis. *Journal of rehabilitation medicine.* 2017;49(1):2-9.

337. Zhu X, Sang L, Wu D, Rong J, Jiang L. Effectiveness and safety of glucosamine and chondroitin for the treatment of osteoarthritis: a meta-analysis of randomized controlled trials. *Journal of orthopaedic surgery and research.* 2018;13(1):170.

338. Zhu X, Wu D, Sang L, et al. Comparative effectiveness of glucosamine, chondroitin, acetaminophen or celecoxib for the treatment of knee and/or hip osteoarthritis: a network meta-analysis. *Clinical and experimental rheumatology.* 2018;36(4):595-602.

339. Zou L, Wang C, Chen K, et al. The Effect of Taichi Practice on Attenuating Bone Mineral Density Loss: A Systematic Review and Meta-Analysis of Randomized Controlled Trials. *International journal of environmental research and public health.* 2017;14(9).

340. Zuo XH, Zhu XP, Bao HG, et al. Network meta-analysis of percutaneous vertebroplasty, percutaneous kyphoplasty, nerve block, and conservative treatment for nonsurgery options of acute/subacute and chronic osteoporotic vertebral compression fractures (OVCFs) in short-term and long-term effects. *Medicine.* 2018;97(29):e11544.
